# *De novo* glycan sequencing by electronic excitation dissociation MS^2^-guided MS^3^ analysis on an Omnitrap-Orbitrap hybrid instrument†

**DOI:** 10.1039/d3sc00870c

**Published:** 2023-06-02

**Authors:** Juan Wei, Dimitris Papanastasiou, Mariangela Kosmopoulou, Athanasios Smyrnakis, Pengyu Hong, Nafisa Tursumamat, Joshua A. Klein, Chaoshuang Xia, Yang Tang, Joseph Zaia, Catherine E. Costello, Cheng Lin

**Affiliations:** a Shanghai Jiao Tong University 800 Dongchuan Road Shanghai 200240 China; b Center for Biomedical Mass Spectrometry, Boston University Chobanian & Avedisian School of Medicine Boston MA 02118 USA chenglin@bu.edu; c Fasmatech Science and Technology 15310 Athens Greece; d Department of Computer Science, Brandeis University Waltham MA 02454 USA; e Department of Chemistry, Boston University Boston MA 02215 USA

## Abstract

Comprehensive *de novo* glycan sequencing remains an elusive goal due to the structural diversity and complexity of glycans. Present strategies employing collision-induced dissociation (CID) and higher energy collisional dissociation (HCD)-based multi-stage tandem mass spectrometry (MS^*n*^) or MS/MS combined with sequential exoglycosidase digestions are inherently low-throughput and difficult to automate. Compared to CID and HCD, electron transfer dissociation (ETD) and electron capture dissociation (ECD) each generate more cross-ring cleavages informative about linkage positions, but electronic excitation dissociation (EED) exceeds the information content of all other methods and is also applicable to analysis of singly charged precursors. Although EED can provide extensive glycan structural information in a single stage of MS/MS, its performance has largely been limited to FTICR MS, and thus it has not been widely adopted by the glycoscience research community. Here, the effective performance of EED MS/MS was demonstrated on a hybrid Orbitrap-Omnitrap QE-HF instrument, with high sensitivity, fragmentation efficiency, and analysis speed. In addition, a novel EED MS^2^-guided MS^3^ approach was developed for detailed glycan structural analysis. Automated topology reconstruction from MS^2^ and MS^3^ spectra could be achieved with a modified GlycoDeNovo software. We showed that the topology and linkage configurations of the Man_9_GlcNAc_2_ glycan can be accurately determined from first principles based on one EED MS^2^ and two CID-EED MS^3^ analyses, without reliance on biological knowledge, a structure database or a spectral library. The presented approach holds great promise for autonomous, comprehensive and *de novo* glycan sequencing.

## Abstract 2

EED MS^2^ spectra were used to generate candidate topologies and guide selection of fragment(s) for MS^3^ analysis. Isotoplogies indistinguishable at the MS^2^ level may be differentiated by CID-EED MS^3^ analysis.

## Graphical abstract

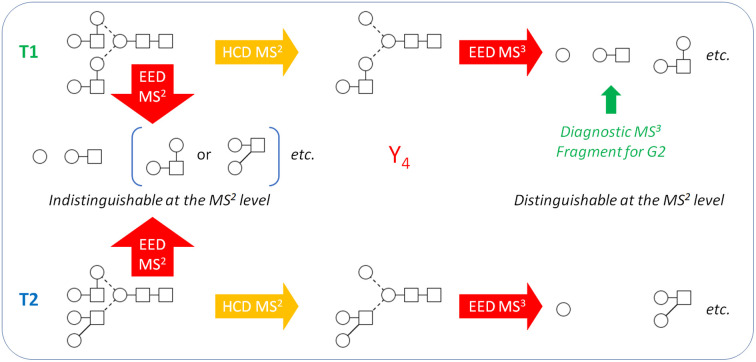

## Introduction

Glycosylation, a diverse and complex post-translational modification, plays vital roles in many biological processes.^1–5^ Elucidation of glycan structures presents considerable challenges due to their structural complexity and heterogeneity, requiring analytical tools that can provide detailed information on their branching patterns, linkages, and stereochemical configurations. Database searching methods are limited to the identification of previously characterized glycans as there can be no genome-predicted glycan database because of the non-template-driven nature of glycan biosynthesis. Thus, discovery of novel glycan structures must be achieved by *de novo* sequencing. Though NMR can provide detailed structural information for glycans,^6^ it typically requires milligrams of purified sample, an amount that is not usually available from biological sources. Tandem mass spectrometry (MS/MS) has been effectively applied for glycan characterization owing to its high sensitivity, specificity, and ease of implementation with on-line separation methods.^7–9^ Although tandem MS analysis is commonly performed with collision-induced dissociation (CID) and higher energy collisional dissociation (HCD), glycan analysis by collision-based MS/MS is characterized by preferential cleavage of glycosidic bonds with few linkage-defining cross-ring fragments, and frequent losses of labile modifications. Consequently, a single stage of CID MS/MS analysis often fails to provide sufficient structural details, and sequential tandem mass spectrometry (MS^*n*^) is usually needed to determine the glycan branching pattern and linkages, and to differentiate structural isomers.^10–17^ However, the MS^*n*^ approach is low-throughput and difficult to automate. Present strategies for automation of the MS^*n*^ process are limited by the availability of glycan structure databases or tandem mass spectral libraries.^18–20^

Major limitations of collision-based MS/MS arise from the slow-heating nature of collisional activation, and may be overcome by employing alternative, radical-driven ion activation methods, such as free radical activated glycan sequencing,^21,22^ ultraviolet photodissociation,^23–25^ charge transfer dissociation,^26^ and various electron-activated dissociation (ExD) methods.^15,27–34^ Among them, electronic excitation dissociation (EED) MS/MS has recently emerged as a powerful tool for structural glycomics. EED can produce detailed structural information in a single stage of MS/MS analysis, and has been successfully implemented with on-line LC and ion mobility separation for effective characterization of glycan mixtures.^34–40^ The power of an integrated approach that combines EED MS/MS with on-line chromatographic separation and software-assisted spectral interpretation was demonstrated in a recent study that employed porous graphitic carbon (PGC)-LC-EED-MS/MS.^40^ Candidate topologies of 18 oligomannose glycans released from RNase B were reconstructed from their EED MS^2^ spectra by GlycoDeNovo,^41^ a *de novo* glycan sequencing software, without reliance on any database. Putative topologies were consistently ranked as the top structures by the software.^40^ Many linkages could also be determined *de novo*, although in some cases, biological insights, such as the glycan biosynthetic rules, were needed to fully define the structure.

To date, glycan structural elucidation by EED MS/MS has only been demonstrated on Fourier transform-ion cyclotron resonance (FTICR) MS instruments. Broad adoption of EED MS/MS by the glycoscience research community has been hindered by the limited accessibility and high operating cost of FTICR MS. Recently, electromagnetostatic cells that may be installed in non-ICR instruments have become commercially available, allowing protein sequencing by electron-based dissociation on Orbitrap, time-of-flight (TOF), and ion trap mass spectrometers.^42–48^ However, effective ExD characterization of glycans on these instruments has yet to be fully realized, in part because ion-electron interaction occurs during analyte transfer through the ExD cell without trapping, and this limits the ExD efficiency, particularly for higher-energy ExD processes. Improved electron activated dissociation (EAD) efficiency may be achieved by trapping the precursor ions for more extensive interactions with electrons.^49^

**Fig. 1:**
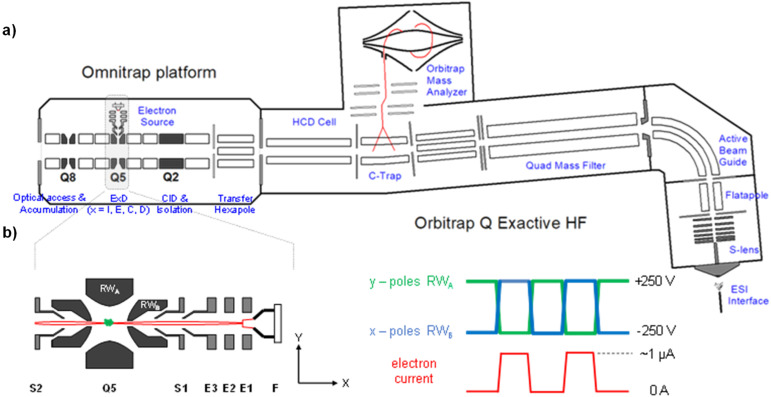
(a) Schematic of the hybrid Omnitrap-Orbitrap platform; (b) principle of operations for ExD analysis performed on the Omnitrap system. The illustration of the ExD cell in (b) is rotated 90 degrees compared to (a).

In this study, we took advantage of a new ExD cell design on a prototype Omnitrap instrument.^50^Fig. 1a shows the schematic of an Omnitrap platform, consisting of a system of linear ion trap segments, connected to the rear of the HCD cell of a QE-HF Orbitrap instrument *via* a transfer hexapole. The segmented ion trap offers flexibilities for ion storage and manipulation, enabling high performance MS^*n*^ analysis by utilizing a wide range of fragmentation methods, including CID and ExD. Interfacing the Omnitrap with an Orbitrap allows subsequent detection of fragment ions with high mass accuracy and resolving power, and this is beneficial for MS/MS-based glycan structural analysis as isobaric fragments are frequently produced by glycans. The principle of operation for ExD experiments on a QE-Omnitrap instrument is illustrated in Fig. 1b. ExD analysis is carried out in Q5 with an orthogonally mounted electron source consisting of a tantalum disc cathode and electrostatic focusing lenses. Electrons are injected into Q5 when a positive potential is applied to the X-electrodes and blocked when a negative potential is applied. The E2 split electrode provides a deflection pulse to ensure electron injection only during the correct RF phase. Unlike conventional ion traps that employ sinusoidal RF waveforms, rectangular waveforms are applied to the Omnitrap electrodes. The rectangular waveform produces a “static’ trapping field, allowing precise control of the electron energy. Time-controlled ion-electron interaction is possible due to analyte trapping in Q5 during the entire ExD event, and this should lead to improved ExD efficiencies.

Here, we report, for the first time, efficient detailed glycan structural characterization by EED MS/MS on a hybrid QE-Omnitrap instrument. To address the challenges in *de novo* glycan sequencing by MS^2^, we developed a novel EED MS^2^-guided MS^3^ strategy to differentiate iso-topologies and to eliminate ambiguities in linkage assignment among different antennae. Additionally, the GlycoDeNovo software was modified to facilitate automated interpretation of both MS^2^ and MS^3^ spectra. The potential of this approach for *de novo* glycan sequencing is demonstrated for characterization of the canonical Man_9_ chitobiose structure (Man_9_GlcNAc_2_) and the biantennary G2 glycan.

## Experimental

### Materials

The Man_9_GlcNAc_2_ and G2 glycan standards were purchased from ProZyme-Agilent (Hayward, MA). HPLC grade water, acetonitrile (CAN), chloroform, and sodium acetate were obtained from Fisher Scientific (Pittsburgh, PA). Micro bio-spin columns (0.8 mL bed volume) were acquired from Bio-Rad Laboratories (Hercules, CA). Methyl iodide, dimethyl sulfoxide (DMSO), sodium hydroxide beads (20–40 mesh), sodium borodeuteride (NaBD_4_), and acetic acid were purchased from Sigma-Aldrich (St. Louis, MO).

### Sample preparation

All glycans were deutero-reduced and permethylated according to protocols described in detail elsewhere.^36,51^ Briefly, 2 μg of glycan was dissolved in 200 μL of NaBD_4_ (250 mM) in 100 mM NH_4_OH solution. Following a 2 h incubation at room temperature, the reaction was quenched by gradual addition of 10% acetic acid until bubbling ceased. Excess NaBD_4_ was removed with addition of methanol. For solid-phase permethylation, an empty spin column was filled with suspended NaOH beads and conditioned with 400 μL of DMSO. Deutero-reduced glycans were dissolved in 120 μL of DMSO plus 5 μL of H_2_O and loaded onto the NaOH column. An aliquot of 100 μL of methyl iodide was added to the column, and the spin-column was settled for 1.5 h, with additions of 100 μL methyl iodide every 30 min. Permethylated glycans were extracted by chloroform and dried with a SpeedVac system (ThermoFisher Scientific, Waltham, MA).

### Mass spectrometry analysis

Deutero-reduced and permethylated glycans were dissolved in 50 : 50 H_2_O : ACN solution containing 100 μM sodium acetate, to a concentration of 1 or 5 pmol μL^−1^, for MS analysis on QE-Omnitrap or FTICR MS, respectively. All QE-Omnitrap experiments were performed on a Q Exactive HF instrument (Thermo Scientific, Bremen, Germany) modified with an Omnitrap (Fasmatech, Athens, Greece). The Omnitrap platform is equipped with an ExD source consisting of a tantalum disc (1.6 mm diameter) and a series of electrostatic focusing lenses to guide electrons into the Q5 segment (Fig. 1). A TriVersa NanoMate nanoESI source (Advion, Ithaca, NY) was used for sample introduction into the mass spectrometer, with an injection time of 5 ms for MS^2^ and up to 300 ms for MS^3^ analyses. For EED MS/MS, precursors were selected by the QE quadrupole, and sent to Q5 in the Omnitrap where they were irradiated with 17–20 eV electrons for 50 ms. For CID-EED MS^3^ analysis, CID was conducted in the Omnitrap Q2 with pulsed argon gas (10 ms dipolar excitation). The CID fragment of interest was isolated in Q2 and sent to Q5 for EED fragmentation, followed by the transfer of product ions back to the Orbitrap for mass analysis. HCD (50 eV) experiments were performed in the QE HCD cell. All spectra were acquired with 5 microscans and a fixed resolving power of 60 K at *m*/*z* 200.

A comparative study was performed on a 12T solariX FTICR MS equipped with a hollow cathode dispenser (Bruker Daltonics, Bremen, Germany). Samples were loaded into a pulled fused silica capillary and directly infused into the mass spectrometer by nano-electrospray. Targeted ions were isolated by the front-end quadrupole and accumulated in an external hexapole collision cell for up to 400 ms. For EED MS/MS analysis, precursor ions were irradiated by 18–20 eV electrons for 100–300 ms. For MS^3^ analysis, quadrupole-selected precursors were accumulated and fragmented by CID in the collision cell, and fragments were sent to the ICR cell and selected by an in-cell sweep isolation for EED MS^3^ analysis. Mass spectra were acquired with a low-mass cutoff of *m*/*z* 200, and a 512k or 1 M data size, resulting in a transient length of 288 ms or 577 ms, and an estimated resolving power of 66 K or 130 K at *m*/*z* 400, respectively. Five and forty spectra were averaged for EED MS/MS and CID-EED MS^3^ analyses, respectively.

### Data analysis

The QE-Omnitrap mass spectral data were processed by Xcalibur and FreeStyle (Thermo Scientific, Bremen, Germany). Peak lists exported from FreeStyle were deconvoluted using ms_deisotope and subsequently interpreted by GlycoDeNovo. FTICR spectra were processed by DataAnalysis 4.4 (Bruker, Bremen, Germany), and peak picking was performed using the SNAP algorithm. Fragments were annotated according to the Domon and Costello nomenclature.^52^ Manual spectra interpretation was assisted by GlycoWorkbench 2.^53^ Candidate topologies were automatically reconstructed by GlycoDeNovo and ranked by IonClassifier.^41^

## Results and discussion

We first compared the performance of EED MS^2^ on two instrument platforms, FTICR MS and QE-Omnitrap MS. For FTICR MS analysis, the reduced and permethylated Man_9_GlcNAc_2_ glycan solution (5 pmol μL^−1^) was loaded into a pulled glass capillary, and directly infused into the mass spectrometer using nano-electrospray ionization. For QE-Omnitrap analysis, the same batch of the derivatized Man_9_GlcNAc_2_ glycan was diluted to a concentration of 1 pmol μL^−1^, and directly infused using an Advion TriVersa NanoMate ESI source. Instrumental parameters were optimized to achieve the highest EED efficiency.

**Fig. 2:**
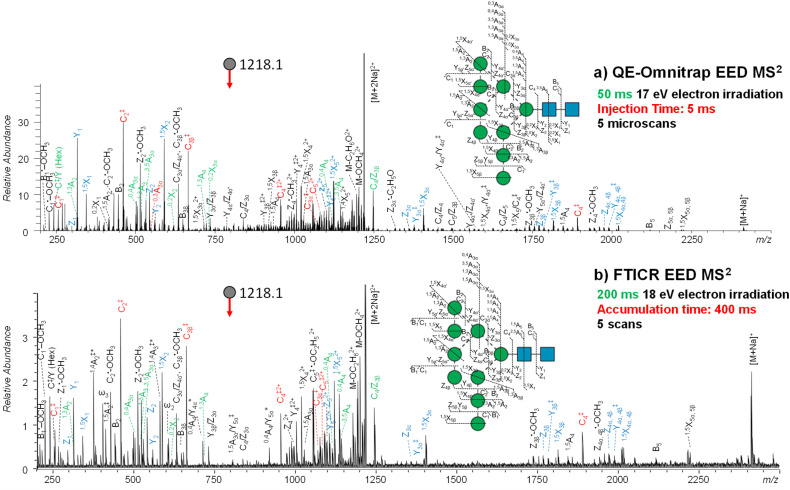
EED MS^2^ spectra and cleavage maps of reduced- and permethylated Man_9_GlcNAc_2_ ([M + 2Na]^2+^ at *m*/*z* 1218.1042) acquired on (a) QE-Omnitrap MS and (b) FTICR MS, respectively. Each EED MS^2^ spectrum was the summed result of 5 scans. A complete series of C-ions and Y/Z/^1,5^X triplets are labeled in red and blue, respectively. Linkage-diagnostic cross-ring and internal fragments are labeled in green. Asterisk indicates singly charged fragments with two sodium atoms. A complete list of assigned fragments can be found in ESI Tables S1 and S2.†

Fig. 2 shows the EED MS^2^ spectra of the doubly-sodiated Man_9_GlcNAc_2_ precursor (*m*/*z* 1218.1042) acquired on the QE-Omnitrap and on the FTICR MS, with all assigned fragments listed in ESI Tables S1 and S2,† respectively. EED produced very similar fragmentation patterns on these two instruments, while the EED efficiency achieved on QE-Omnitrap was significantly higher than on FTICR MS, producing on average 5–10 times higher relative fragment abundance and signal-to-noise ratio. With a higher S/N ratio, 31 more peaks were assigned in the QE-Omnitrap EED MS^2^ spectrum than in the corresponding FTICR spectrum, representing a 25% increase in the number of assigned peaks. Notably, the Omnitrap spectrum of the five-fold diluted sample solution was acquired with a shorter ion injection time (5 ms *vs.* 400 ms) and electron irradiation time (50 ms *vs.* 200 ms) than the ICR spectrum. The shorter spectral acquisition time and higher EED efficiency achieved on QE-Omnitrap make it far more suitable for high-throughput LC-MS/MS analysis, allowing characterization of a higher number of glycoforms, including those present in lower abundance. The higher EED efficiency achieved on the QE-Omnitrap instrument may have benefited from collisional cooling and focusing in the higher-pressure Q5 that improves the electron–ion interaction, whereas in the ultra-high vacuum of the ICR cell, axial excitation and magnetron expansion can lead to poorer ion-electron overlap.^54^

**Fig. 3:**
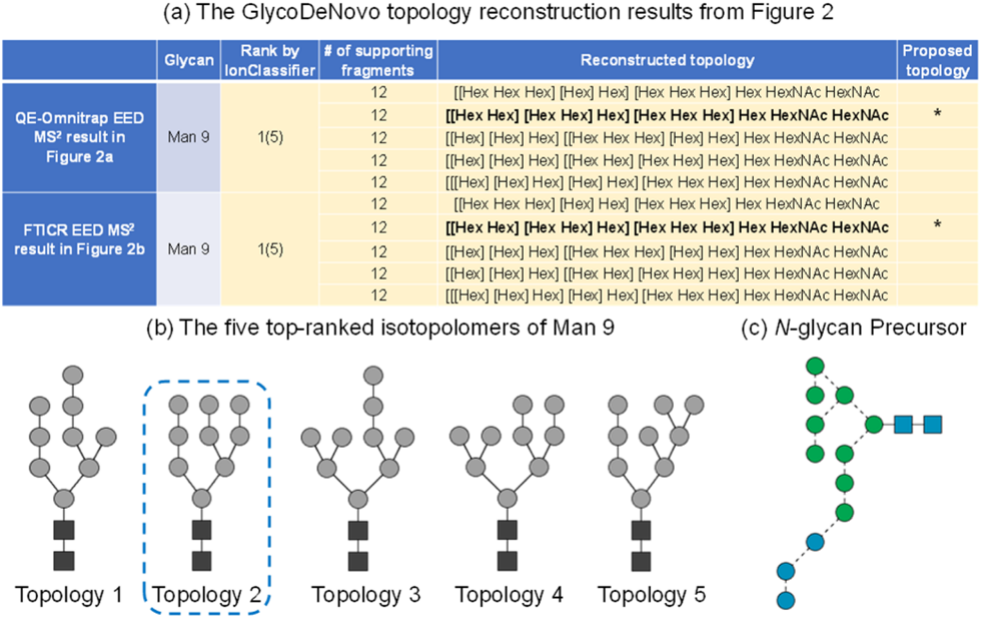
(a) Top-ranked candidate topologies reconstructed by GlycoDeNovo from the EED MS^2^ spectrum of deutero-reduced and permethylated Man_9_GlcNAc_2_ in Fig. 2; (b) graphic representations of the five top-ranked isotopolomers of Man_9_GlcNAc_2_; (c) SNFG representation of the *N*-glycan precursor.

On either instrument platform, EED of the Man_9_GlcNAc_2_ glycan produced a complete series of C-type ions (C1/C1‡, C2/C2‡, C3β/C3β‡, C3α/C3α‡, C4‡, C5‡, where the symbol ‡ indicates loss of two hydrogens), and Y/Z/^1,5^X triplets. The presence of these sequence ions allows *de novo* reconstruction of the Man_9_GlcNAc_2_ candidate topologies by GlycoDeNovo. The canonical structure, listed in bold in Fig. 3a with its topology encircled in Fig. 3b, is one of the five candidates with the highest IonClassifier score. The IonClassifier was established by a machine learning approach that identifies common spectral context of a certain type of fragment in a training data set, defined as a collection of its related neighboring peaks, represented by their respective mass shifts and relative abundances. The IonClassifier can be used to evaluate the accuracy of an assignment by examining the spectral context of the fragment.^41^ The IonClassifier score of a candidate topology is the sum of the IonClassifier scores of all its supporting peaks, namely, glycosidic fragments consistent with the topology. Here, the five top-ranked topologies are indistinguishable at the MS^2^ level, as they produce the same set of supporting peaks, including non-reducing-end glycosidic fragments with compositions of Hex, Hex_2_, Hex_3_, Hex_5_, Hex_9_, and Hex_9_HexNAc. Previously, the correct Man_9_GlcNAc_2_ topology was assigned based on prior glycan biosynthetic knowledge.^40^ Among the five top-ranked candidates, only topology 2, with a linear tri-hexose branch and a branched penta-hexose branch, can be derived from the structure of the tetradecasaccharide *N*-linked glycan precursor (Fig. 3c).

Once a topology is defined, most linkages can be independently determined based on linkage-diagnostic fragments without further reference to the precursor structure. For example, the presence of ^3,5^A_4_ (*m*/*z* 1145.5566), ^0,4^A_4_ (*m*/*z* 1117.5256), and C_4_/Z_3β_ (*m*/*z* 1247.5889) ions not only places the pentasaccharide branch to the 6-antenna, but also localizes the tri-hexose branch to the 3-antenna, since a high-abundance C/Z ion is associated with the loss of the C3-substituent from a Z ion. The observation of ^1,3^A_4_ (*m*/*z* 723.3408) and ^0,2^X_2_ (*m*/*z* 618.3319) ions also corroborates with the assignment of the tri-hexose to the 3-antenna. Within the 6-antenna, the presence of ^0,4^A_3α_ (*m*/*z* 505.2253), ^3,5^A_3α_ (*m*/*z* 533.2568), C_3α_/Z_4α′′_ (*m*/*z* 635.2886), and ^0,2^X_3α_ (*m*/*z* 728.8606) ions is consistent with a penta-hexose structure consisting of two di-hexose branches, at the 6- and 3-positions of the branching mannose, respectively, although the 1→6 linkage assignment is not conclusive, as the observed ^0,4^A_3α_ and ^3,5^A_3α_ ions can theoretically be generated from the linear tri-hexose 3-antenna as well. Similarly, the 1→2 linkage between the second and third mannose residues on the 3-antenna cannot be assigned definitively, since its diagnostic ^1,3^A_3β_ (*m*/*z* 519.2411) ion is isomeric to the ^1,3^A_3α′′_ at the 6-antenna. Finally, while the presence of a ^1,3^A_2_ ion (*m*/*z* 315.1410) and the absence of ^0,2^X_4_, ^3,5^A_2_, and ^0,4^A_2_ ions strongly suggest the existence of at least one 1→2-linked non-reducing-end mannose residue, its location (or their locations) cannot be unambiguously determined. ESI Scheme S1b† shows a hypothetical structure that can produce the same group of cross-ring and internal fragments as the true structure (ESI Scheme S1a†). Detailed discussions on the general EED mechanism and formation of specific diagnostic fragments have been presented elsewhere.^36,37,40,55^

The analysis above clearly illustrates the challenges of MS^2^-based *de novo* glycan sequencing. Even with complete series of glycosidic fragments and cross-ring fragments, it is sometimes not possible to differentiate iso-topologies or accurately determine linkages among different branches. Additional structural details may be revealed by performing sequential tandem MS analysis on MS^2^ fragment(s) of interest. Presently, there are several ways to choose MS^2^ fragments as precursors for later stages of MS^*n*^ analysis, each with its merits and limitations. The most straightforward method is to select product ion(s) of the highest abundance. Although such an approach allows autonomous precursor selection, the highest-abundance fragments do not always produce the most structurally informative MS^3^ spectra. Alternatively, precursors may be selected by expert users, aided by existing knowledge of the glycan fragmentation behavior and/or biological insights.^56–58^ This is typically performed during direct infusion analyses on ion trap instruments, whose low mass resolving power can negatively impact the accuracy of the fragment assignment. Further, without chromatographic separation, each MS^2^ precursor may consist of multiple isomeric structures, and each MS^2^ fragment chosen for further MS^*n*^ analysis can be a mixture of isobaric structures. Consequently, confident structural assignment requires investigation of many gas-phase disassembly pathways, often involving deeper level of MS^*n*^. This process is difficult to automate, and the need to perform MS^*n*>3^ limits both the sensitivity and throughput. Recently, Sun and coworkers developed a glycan intelligent precursor selection (GIPS) strategy to guide MS^*n*^ experiments.^59,60^ GIPS utilizes a statistical model to calculate the distinguishing power of fragments, which dictates the selection of precursor(s) for next stage of tandem MS analyses. The GIPS approach has only been demonstrated for the identification of the glycan branching patterns, but not for linkage determination. Moreover, when calculating the distinguishing power, it only considers structures present in the existing glycan database, and thus it cannot identify new structures.

The MS^*n*^ method may also be combined with spectral library search for glycan structural elucidation, as utilized in logically derived sequence (LODES)/MS^*n*^.^20,61,62^ The LODES/MS^*n*^ approach employs a built-in logical procedure to successively break down an oligosaccharide sequence into its monosaccharide and disaccharide components. The CID spectra of these smaller fragments can then be searched against a spectral database for structural assignment. The LODES/MS^*n*^ approach can provide detailed structural information, provided that the corresponding spectral library of disaccharide standards is available. However, it still requires investigation of many MS^*n*^ pathways, and is not readily compatible with on-line LC-MS analysis. Multiple LC injections were needed to fully define the structure of glycans as small as trisaccharides.^62^ For larger oligosaccharides, such as released *N*-linked glycans, knowledge on their biosynthesis was utilized to reduce the number of MS^*n*^ pathways that need to be examined. For complex mixtures, it was necessary to perform multi-dimensional LC fractionation off-line before LODES/MS^*n*^ analysis.^20^

**Fig. 4:**
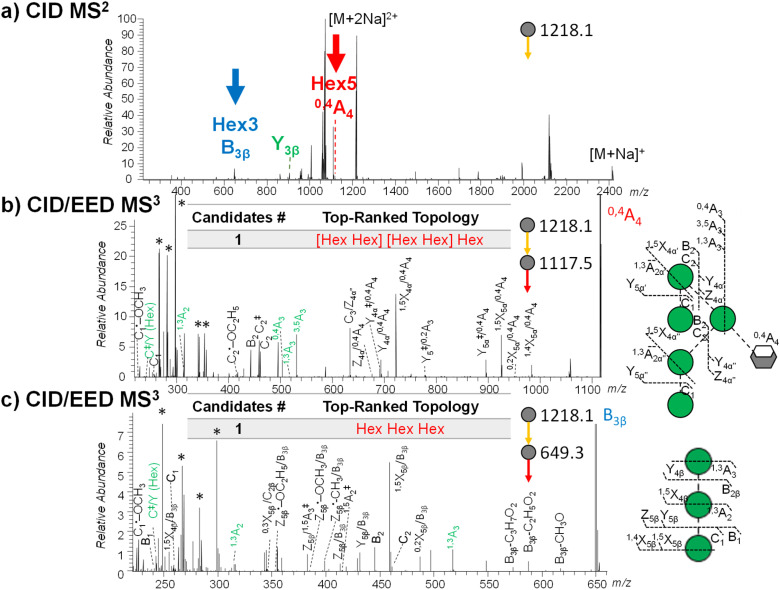
(a) CID MS^2^ spectra of deutero-reduced- and permethylated Man_9_GlcNAc_2_ ([M + 2Na]^2+^ at *m*/*z* 1218.1042) acquired on the QE-Omnitrap system. Fragments selected for EED MS^3^ are labeled. (b) and (c) CID-EED MS^3^ spectra and cleavage maps of the ^0,4^A_4_ (*m*/*z* 1117.5259) and B_3β_ (*m*/*z* 649.3046) ions, respectively, acquired on QE-Omnitrap. Linkage-diagnostic cross-ring and internal fragments are labeled in green. Peaks marked by asterisks are background peaks with mass defects that are not expected from glycan fragments. Complete lists of assigned fragments can be found in ESI Tables S3 and S4.†

Here, precursor(s) for MS^3^ analyses were selected based on their projected differentiating power on top-ranked topology candidates identified by GlycoDeNovo from the EED MS^2^ spectrum. For Man_9_GlcNAc_2_, the top five topologies (Fig. 3b) can be uniquely differentiated by performing MS^3^ analysis on the CID-generated Hex_3_ and Hex_5_ fragments (Fig. 4a). EED of a Hex_5_ fragment, ^0,4^A_4_ at *m*/*z* 1117.5259, produced glycosidic fragments with either one or two Hex units (C_1_ ion at *m*/*z* 259.1162, B_2_ at *m*/*z* 445.2045, and C_2_ at *m*/*z* 463.2153), but not with three or four Hex units (Fig. 3b), hence eliminating topologies 1, 3, and 5 from consideration. The remaining two topologies, 2 and 4, can be distinguished by EED analysis of a Hex_3_ fragment, B_3β_ at *m*/*z* 649.3046. The presence of two Hex_2_ fragments (B_2_ at *m*/*z* 445.2048 and C_2_ at *m*/*z* 463.2162) in the EED spectrum of B_3β_ (Fig. 3c) clearly established topology 2 as the only structure consistent with the MS^3^ results. The same conclusion could be reached by applying a modified GlycoDeNovo algorithm for analysis of the MS^3^ spectra. In this case, the software treated the mass difference between the intact glycan and the MS^2^ fragment as a reducing-end modification. GlycoDeNovo successfully identified a penta-saccharide with two di-hexose branches and a linear tri-hexose structure as the top-ranked topologies for the Hex_5_ and Hex_3_ substructures, respectively. Thus, we showed that the correct topology of the Man_9_GlcNAc_2_ glycan can be defined by just two MS^3^ analyses, autonomously from the first principles, with no need for reference spectral libraries, or biological knowledge. Here, the applicability of EED MS/MS to analysis of singly charged precursor is essential for the CID-EED MS^3^ workflow, as both the ^0,4^A_4_ and B_3β_ fragments selected for MS^3^ analysis were singly charged, and therefore could not be characterized by charge-reducing MS/MS methods, such as ETD and ECD.

It is worth noting that a given substructure may be present in several fragments. For MS^2^ fragments with the same differentiating power, the choice is generally made based on the fragment ion abundance and the ability to achieve a clean isolation. Sometimes, more than one MS^2^ fragment can generate similarly informative MS^3^ spectra. For example, the Y_3β_^2+^ ion at *m*/*z* 904.9469 also retained the Man_5_ substructure without interference from the Man_3_ branch. EED of Y_3β_^2+^ (ESI Fig. S1 and Table S5†) similarly produced non-reducing-end glycosidic fragments with compositions of Hex_1_ or Hex_2_, but not Hex_3_ or Hex_4_, consistent with the branched Hex_5_ substructure assignment based on the EED spectrum of the ^0,4^A_4_ ion.

As discussed earlier, once topology 2 is established as the correct structure, the Hex_5_ and Hex_3_ branches can be accurately located to the 6- and 3-antennae, respectively, on the basis of the characteristic cross-ring and abundant C/Z internal fragments observed in the EED MS^2^ spectrum. Within the 6-antenna, one of the di-hexose branches can be assigned to the 3-position based on the presence of a high-abundance C_3_/Z_4α′′_ ion at *m*/*z* 635.2886, but the location of the other di-hexose branch cannot be confidently assigned due to potential interference by fragments from the linear tri-hexose 3-antenna. With MS^3^, the Hex_5_ substructure can be isolated for accurate linkage analysis. EED MS^3^ of the ^0,4^A_4_ ion generated both the ^0,4^A_3_ ion at *m*/*z* 505.2262 and the ^3,5^A_3_ ion at *m*/*z* 533.2575, thus unambiguously assigning the second di-hexose branch to the 6-position. The presence of the ^1,3^A_2_ ion at *m*/*z* 315.1416 and a C^‡^/Y (Hex) ion at *m*/*z* 243.0844, together with the lack of a ^0,2^X_4β_/^0,4^A_4_ ion at *m*/*z* 751.3359, placed the terminal mannose residues to the 2-position. Similarly, the linear tri-hexose branch at the 3-antenna was isolated in the B_3β_ fragment, whose EED MS^3^ analysis generated ^1,3^A_2_ at *m*/*z* 315.1415, ^1,3^A_3_ at *m*/*z* 519.2409, C^‡^/Y (Hex) ion at *m*/*z* 243.0842, but ^0,2^X_4β_ at *m*/*z* 283.1149, supporting the 1→2 linkages between the mannose residues. Collectively, the linkages within both antennae could be determined *de novo* by the CID-EED MS^3^ analysis.

With the Omnitrap-Orbitrap system, the MS^3^ experiment can be performed in several different configurations. The CID-EED sequence was chosen here as CID typically generates a smaller number of fragments in higher abundance than EED. Thus, the CID spectrum is usually not as congested as the EED spectrum, making it easier to achieve a clean isolation of the high-abundance MS^2^ fragment of interest. For Man_9_GlcNAc_2_, isolation of the ^0,4^A_4_ fragment at *m*/*z* 1117.5256 from EED MS^2^ can be complicated by co-isolation of the doubly charged ^3,5^A_6_ ion at *m*/*z* 1115.0348 (Fig. 2a and Table S1†), whereas the interfering ^3,5^A_6_ ion was absent in the CID spectrum of Man_9_GlcNAc_2_ (Fig. 4a). On the other hand, EED is the preferred final-stage dissociation method, producing MS^3^ spectra with much higher structural information content than CID. The EED and CID MS^3^ cleavage maps of the ^0,4^A_4_ ion are shown in Fig. 4b and ESI Fig. S2,† respectively. It is evident that many linkage-diagnostic fragments, such as ^1,3^A and C^‡^/Y (Hex) ions, were absent in the CID spectrum (Fig. S2†). Finally, although it is possible to perform CID-EED MS^3^ analysis on a hybrid Qh-FTICR MS instrument, selection of the MS^3^ precursor can only be achieved with in-cell isolation, resulting in a much lower ion count for the precursor of interest. Consequently, even with more than 10 times the ion injection time, the CID-EED MS^3^ spectrum of the ^0,4^A_4_ ion on FTICR MS (ESI Fig. S3†) was of much lower quality than that obtained on the Omnitrap-Orbitrap system (Fig. 4b).

**Fig. 5:**
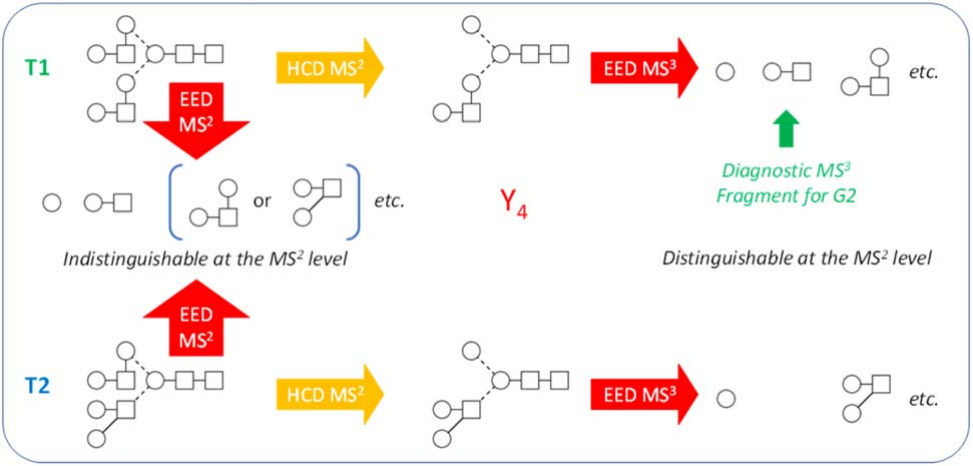
Isotopologies T1 and T2 were indistinguishable by MS^2^ of the G2 glycan, whereas diagnostic HexHexNAc fragments generated by HCD-EED MS^3^ of the Y_4_ ion can be used to define T1 as the correct topology.

While the discussion thus far has been focused on the Man_9_GlcNAc_2_ structure, the workflow presented here can be effectively applied to characterize other glycan structures, including complex-type glycans. Note that the Man_9_GlcNAc_2_ structure was chosen as the primary example not because of its apparent structural simplicity, but rather, because it represents one of the hardest cases for isotopolomer differentiation. With only one type of monosaccharide residue (hexose) present in all branches, many structures can produce the same set of glycosidic fragments and are therefore impossible to differentiate at the MS^2^ level, even with the IonClassifier. Two additional MS^3^ analyses were required to unambiguously define the canonical structure. On the other hand, complex-type glycans consist of a larger variety of monosaccharide building blocks that have different masses and are less likely to produce isotopologies. True topologies of many complex-type glycans can be identified at the MS^2^ level, though there are cases where MS^3^ is necessary. Fig. 5 shows one such case for characterization of the biantennary G2 glycan. At the MS^2^ level, topologies 1 and 2 (T1 and T2) would produce the same set of glycosidic fragments (Hex, HexHexNAc, Hex_2_HexNAc, *etc.*), and were co-ranked as the top candidate by the IonClassifier based on the EED MS/MS spectrum of the deutero-reduced and permethylated G2 glycan (ESI Fig. S4†). These two topologies could be differentiated based on the HCD-EED MS^3^ spectrum of the Y_4_ ion (ESI Fig. S5†), where the presence of HexHexNAc fragments and their complementary ions supported T1 as the correct topology. Lists of assigned peaks for the EED spectra of the G2 glycan and its Y_4_ fragment can be found in ESI Tables S6 and S7.†

## Conclusions

The recently introduced hybrid Omnitrap-Orbitrap system allows EED MS^2^ analysis of glycans to be performed with high sensitivity, fragmentation efficiency, and spectral acquisition rate that are crucial for high-throughput analysis. Candidate topology structures can be automatically reconstructed and ranked by the GlycoDeNovo software based on the EED MS^2^ spectrum. Top-ranked topology candidates identified by EED MS^2^ can then be used to guide the selection of MS^2^ fragments for MS^3^ analysis. With one EED MS^2^ and two CID-EED MS^3^ spectra, the complete structure of even the particularly challenging example, Man_9_GlcNAc_2_, including its topology and linkages, could be confidently assigned *de novo* without the need for biological knowledge or a spectral library. With high sensitivity and analysis speed, the novel EED MS^2^-guided-MS^3^ approach presented here should be fully compatible with on-line LC separation, and holds great promise for automated, *de novo*, and comprehensive glycan structural elucidation.

## Data availability

All mass spectral data are included in the ESI.†

## Author contributions

JW and CL conceptualized the experimental design. JW and CX prepared the glycan samples for analysis. DP, MK, and AS performed the initial analysis on the Omnitrap system. JW performed the comparative study on FTICR MS. CX and CL performed additional analysis on the Omnitrap-Orbitrap system. JW, NT, and CL did the data interpretation. JAK assisted with spectral deconvolution by ms_deisotope. PH performed automated spectral interpretation by GlycoDeNovo. JW and CL co-wrote the manuscript. All authors contributed to the editing and revision of the manuscript.

## Conflicts of interest

DT, MK, and AS are affiliated with Fasmatech, the inventor and manufacturer of the Omnitrap instrument.

## Supplementary Material

SC-014-D3SC00870C-s001

SC-014-D3SC00870C-s002
