# Supplementary material for: De novo glycan sequencing by electronic excitation dissociation MS2-guided MS3 analysis on an Omnitrap-Orbitrap hybrid instrument
Source: Chem Sci. 2023 Jun 2;14(24):6695–704. doi: 10.1039/d3sc00870c (PMC10284134; doi:10.1039/d3sc00870c)
Supplement: SC-014-D3SC00870C-s001 [file SC-014-D3SC00870C-s001.pdf]

Electronic Supplementary Material (ESI) for Chemical Science.
This journal is © The Royal Society of Chemistry 2023

Supporting Information for:

De Novo Glycan Sequencing by Electronic Excitation Dissociation MS^2-Guided MS^3 Analysis on an Omnitrap-Orbitrap Hybrid Instrument

Juan Wei,[1,2] Dimitris Papanastasiou,[3] Mariangela Kosmopoulou,[3] Athanasios Smyrnakis,[3] Pengyu Hong,[4] Nafisa Tursumamat,[1] Joshua A. Klein,[2] Chaoshuang Xia,[2] Yang Tang,[2,5] Joseph Zaia,[2] Catherine E. Costello,[2,5] Cheng Lin[2]

1. Shanghai Jiao Tong University, Shanghai, 800 Dongchuan Road, Shanghai, 200240, China
2. Center for Biomedical Mass Spectrometry, Boston University Chobanian & Avedisian School of Medicine, Boston, MA 02118, United States
3. Fasmatech Science and Technology, 15310 Athens, Greece
4. Department of Computer Science, Brandeis University, Waltham, MA 02454, United States
5. Department of Chemistry, Boston University, Boston, MA 02215, United States

Corresponding Authors

Cheng Lin − Center for Biomedical Mass Spectrometry, Boston University Chobanian and Avedisian School of Medicine, Boston, Massachusetts 02118, United States; orcid.org/0000-0003-3653-9633

Phone: 1-617-358-2428

Email: chenglin@bu.edu

Fax: 617-358-2416

Juan Wei − School of Pharmacy, Shanghai Jiao Tong University, 800 Dongchuan Road, Shanghai 200240, China

Phone: 86-21-3420-4048

Email: wwwjuan@sjtu.edu.cn

Table of Contents

**Scheme S1**	Page S-3
**Figure S1**	Page S-3
**Figure S2**	Page S-4
**Figure S3**	Page S-4
**Figure S4**	Page S-5
**Figure S5**	Page S-6
**Table S1**	Page S-7
**Table S2**	Page S-12
**Table S3**	Page S-16
**Table S4**	Page S-18
**Table S5**	Page S-20
**Table S6**	Page S-23
**Table S7**	Page S-27

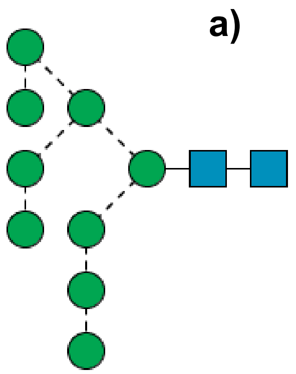

Scheme S1. SNFG representation of (a) the canonical $Man_9GlcNAc_2$ structure, and (b) a hypothetical structure that can generate the same group of cross-ring and internal fragments as the true structure in (a).

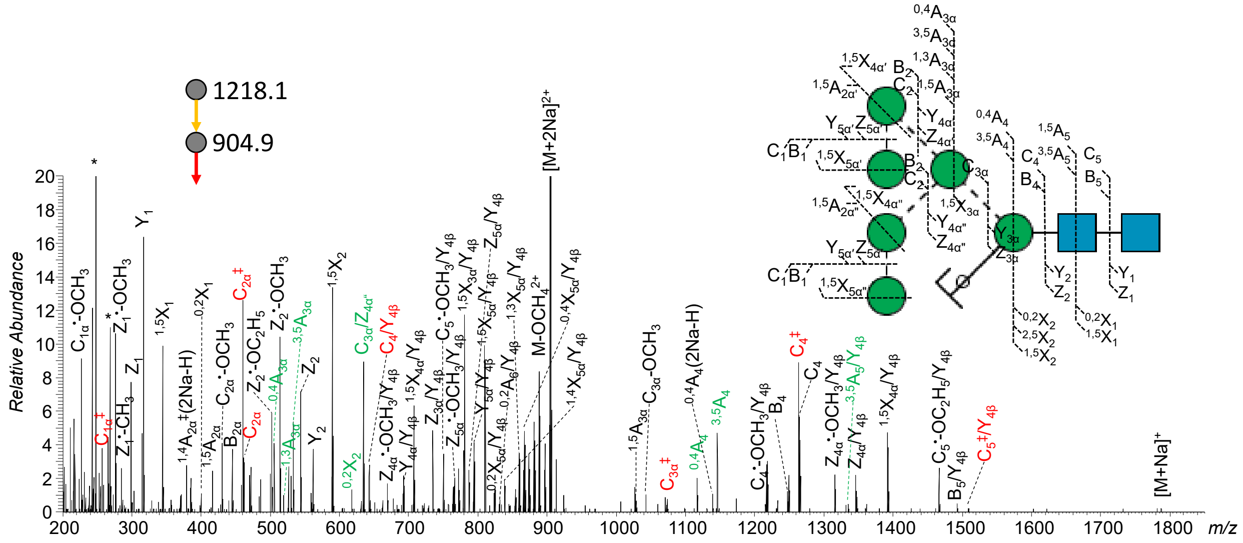

Figure S1. The QE-Omnitrap CID-EED MS³ spectrum of the $Y_{3\beta}^{2+}$ ion (*m/z* 904.9469) of deutero-reduced and permethylated $Man_9GlcNAc_2$. Inset shows the cleavage map. An ion injection time of 20 ms and an electron irradiation time of 50 ms were used. The spectrum was acquired with 10 microscans. The complete series of glycosidic C-ions are labeled in red; linkage-diagnostic cross-ring and internal fragments are labeled in green. Asterisk denotes noise peaks with mass defects inconsistent with glycan fragments. A complete list of assigned fragments can be found in Supporting Table S5.

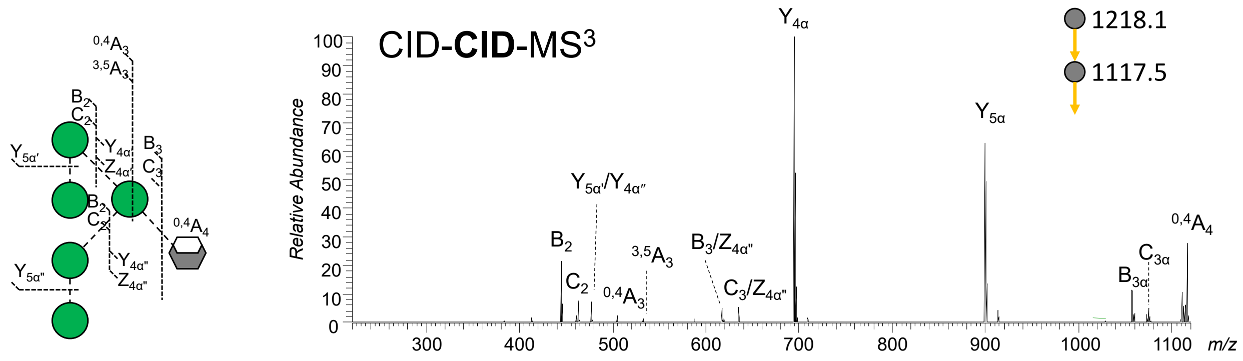

Figure S2. The QE-Omnitrap CID-CID MS^3 spectrum and the cleavage map of the CID fragment from deutero-reduced and permethylated $Man_9GlcNAc_2$ at *m/z* 1117.5259 ($^{0,4}A_4$).

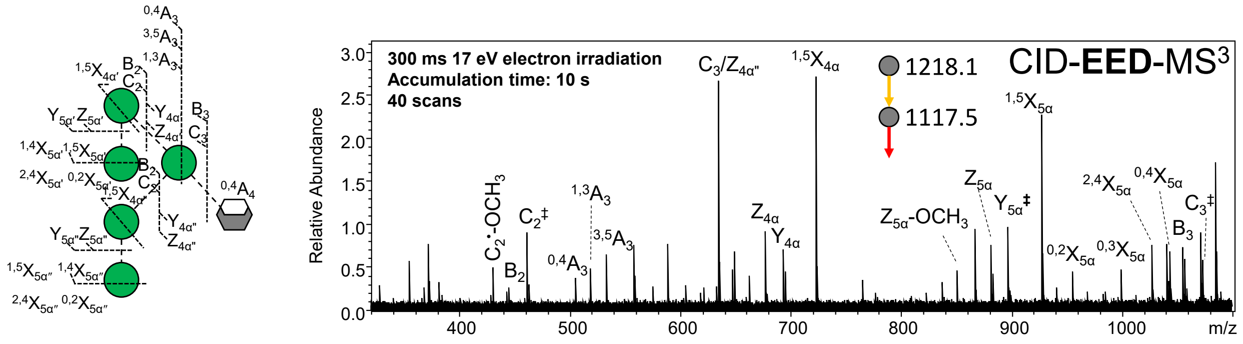

Figure S3. The FTICR CID-EED MS^3 spectrum and the cleavage map of the CID fragment from deutero-reduced and permethylated $Man_9GlcNAc_2$ at *m/z* 1117.5259 ($^{0,4}A_4$).

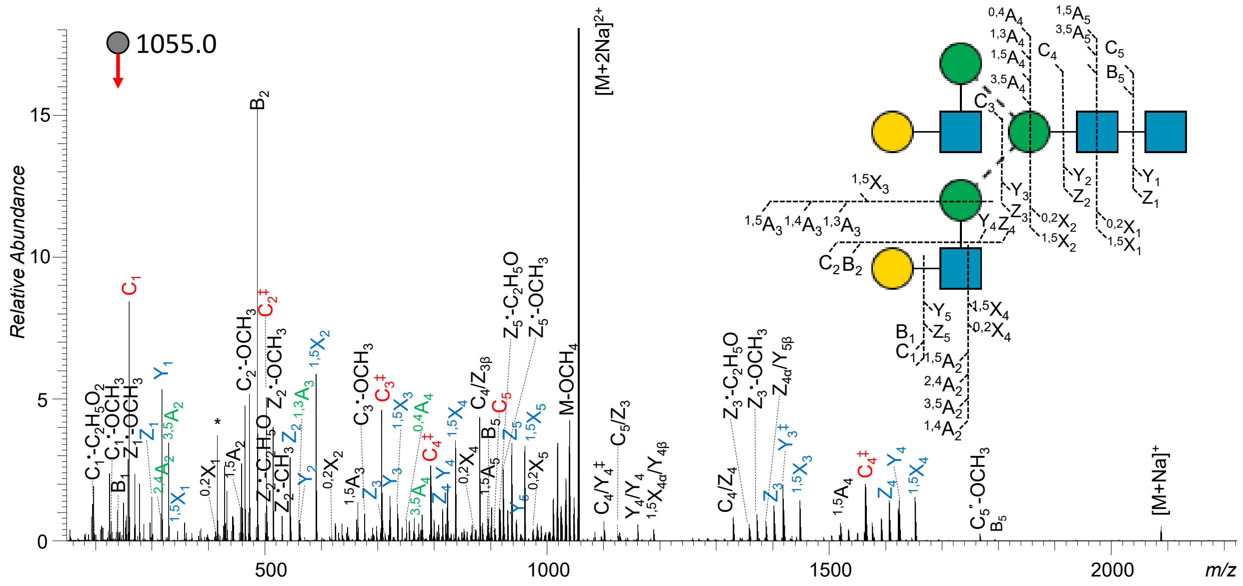

Figure S4. EED MS^2 spectrum and cleavage map of reduced- and permethylated G2 ($Hex_5HexNAc_4$, $[M+2Na]^{2+}$ at *m/z* 1055.0308) acquired on QE-Omnitrap MS. A complete series of C-ions and Y/Z/^{1,5}X triplets are labeled in red and blue, respectively. Linkage-diagnostic cross-ring and internal fragments are labeled in green. A complete list of assigned fragments can be found in Supporting Tables S6.

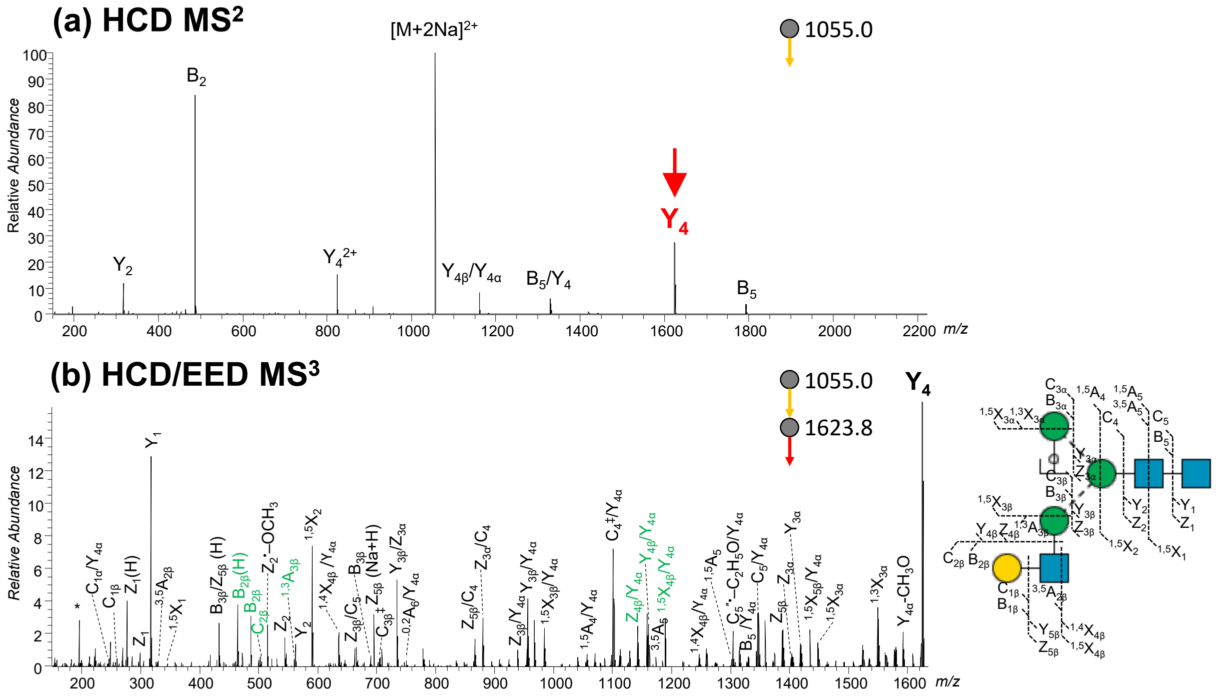

Figure S5. a) HCD (50 eV) MS^2 spectrum of reduced- and permethylated G2 ($Hex_5HexNAc_4$, $[M+2Na]^{2+}$ at *m/z* 1055.0315) acquired on QE-Omnitrap MS. The Y_4 ion highlighted was selected for MS^3 analysis. b) HCD-EED MS^3 spectrum of the Y_4 ion at *m/z* 1623.8311 acquired on QE-Omnitrap MS. The maximum ion injection time was 300 ms, and the electron (20 eV) irradiation time was 50 ms. The top-ranked topology of the EED MS^3 spectrum analyzed by GlycoDeNovo software is inserted correspondingly. All ions were detected in the orbitrap. Fragments diagnostic to the canonical G2 structure are labeled in green. A complete list of assigned fragments can be found in Supporting Tables S7.

Table S1. List of assigned fragments by EED MS^2 of deutero-reduced and permethylated $Man_9GlcNAc_2$ ($[M+2Na]^{2+}$ at *m/z* 1218.1042), acquired on the QE-Omnitrap instrument (spectrum shown in Figure 2a). All fragments are sodium adducts unless labeled otherwise. Multiple assignments are separated by commas; the double dagger symbol (‡) indicates that the fragment has two fewer hydrogens than the canonical structures; the double prime symbol (") indicates that the fragment has two more hydrogens than the canonical structure; the dot symbol (•) indicates a radical.

z	Exp. *m/z*	Theo. *m/z*	Assignment	Δ, ppm
1	197.0783	197.0783	$C_1^{\bullet}$-C_2H_5O	0.00
1	211.0939	211.0941	$B_1^{\bullet}$-CH_3O	-0.95
1	227.0888	227.0890	$C_1^{\bullet}$-CH_3O	-0.88
1	243.0838	243.0839	C‡/Y (Hex)	-0.41
1	253.1267	253.1269	$Z_1^{‡\bullet}$-C_2H_5O	-0.79
1	255.1422	255.1424	$Z_1^{\bullet}$-C_2H_5O	-0.78
1	257.0993	257.0996	$C_1^{‡}$	-1.17
1	259.1150	259.1152	C_1	-0.77
1	269.1579	269.1582	$Z_1^{\bullet}$-CH_3O	-1.11
1	296.1101	296.1105	B_5/$^{1,5}X_1$	-1.35
1	299.1686	299.1687	Z_1	-0.33
1	313.1257	313.1258	$^{1,3}A_2^{‡}$	-0.32
1	315.1043	315.1050	$C_2^{‡}$/$^{2,5}X_5$	-2.22
1	315.1410	315.1414	$^{1,3}A_2$	-1.27
1	315.1632	315.1637	$Y_1^{‡}$	-1.59
1	317.1791	317.1793	Y_1	-0.63
1	329.1204	329.1207	$C_2^{‡}$/$^{1,4}X_5$	-0.91
1	329.1570	329.1571	$^{1,4}A_2$-CH_3O	-0.30
1	345.1740	345.1742	$^{1,5}X_1$	-0.58
1	357.1514	357.1520	$^{1,4}A_2^{‡}$	-1.68
1	371.1674	371.1676	$B_{2\beta}$/$^{0,4}X_{5\beta}$	-0.54
1	401.1781	401.1781	$C_2^{\bullet}$-$C_2H_5O_2$	0.00
1	403.2156	403.2161	$^{1,4}X_1$	-1.24
1	413.1779	413.1782	$B_{3\beta}$/$Z_{5\beta}$	-0.73
1	414.2320	414.2321	$^{0,2}X_1$	-0.24
1	415.1936	415.1939	$^{1,5}A_2^{‡}$	-0.72
1	417.1730	417.1732	$C_2^{\bullet}$-C_2H_5O	-0.48
1	417.2092	417.2095	$^{1,5}A_2$	-0.72

z	Exp. *m/z*	Theo. *m/z*	Assignment	Δ, ppm
1	429.1731	429.1731	$C_2^{\ddagger\bullet}$-CH_3O	0.00
1	431.1885	431.1888	$C_2^{\bullet}$-CH_3O	-0.70
1	445.2042	445.2044	B_2	-0.45
1	447.1834	447.1837	$C^{\ddagger}_{3\beta}/Y_{5\beta}$	-0.67
1	458.2580	458.2583	$^{0,3}X_1$	-0.65
1	459.1836	459.1837	$B_{3\beta}/^{1,5}X_{5\beta}$	-0.22
1	461.1990	461.1993	$C_2^{\ddagger}$	-0.65
1	463.2158	463.2150	C_2	1.73
1	475.1783	475.1786	$C_{3\beta}^{\ddagger}/^{1,5}X_{5\beta}$	-0.63
1	484.2737	484.2740	$Z_2^{\bullet}$-$C_2H_5O_2$	-0.62
1	487.2148	487.2150	$Z_{5\beta}/^{1,3}A_{3\beta}$	-0.41
1	500.2687	500.2688	$Z_2^{\bullet}$- C_2H_5O	-0.20
1	502.2844	502.2845	$Z_2^{\prime\prime\bullet}$- C_2H_5O	-0.20
1	503.2094	503.2099	$^{0,4}A_{3\alpha}^{\ddagger}$	-0.99
1	505.2253	505.2255	$^{0,4}A_{3\alpha}$	-0.40
1	514.2845	514.2845	$Z_2^{\bullet}$-CH_3O	0.00
1	517.2253	517.2255	$^{1,3}A_{3\beta, 3\alpha}^{\ddagger}$	-0.39
1	519.2411	519.2412	$^{1,3}A_{3\beta, 3\alpha}$	-0.19
1	530.2796	530.2794	$Z_2^{\bullet}$-CH_3	0.38
1	531.2415	531.2412	$^{3,5}A_{3\alpha}^{\ddagger}$	0.56
1	533.2568	533.2568	$^{3,5}A_{3\alpha}$	0.00
1	544.2950	544.2951	Z_2	-0.18
1	547.2364	547.2361	$^{0,3}A_{3\alpha}^{\ddagger}$	0.55
1	549.2521	549.2518	$^{0,3}A_{3\alpha}$	0.55
1	560.2924	560.2900	$Y_2^{\ddagger}$	4.28
1	561.2524	561.2518	$^{1,4}A_{3\beta, 3\alpha}^{\ddagger}$	1.07
1	562.3060	562.3056	Y_2	0.71
1	563.2672	563.2674	$^{1,4}A_{3\beta, 3\alpha}$	-0.36
1	575.2677	575.2674	$B_{3\beta}/^{0,4}X_{5\beta}$	0.52
1	590.3009	590.3005	$^{1,5}X_2$	0.68
1	605.2782	605.2779	$C_{3\beta}^{\bullet}$-$C_2H_5O_2$	0.50
1	618.3319	618.3318	$^{0,2}X_2$	0.16
1	621.2731	621.2730	$C_{3\beta}^{\bullet}$-C_2H_5O	0.16
1	621.3089	621.3093	$^{1,5}A_{3\beta}$	-0.64
1	633.2728	633.2729	$C_{3\beta}^{\ddagger\bullet}$-$CH_3O$, $C_{3\alpha}^{\ddagger}/Z_{4\alpha''}$	-0.16
1	635.2886	635.2885	$C_{3\beta}^{\bullet}$-CH_3O, $C_{3\alpha}/Z_{4\alpha''}$	0.16
1	649.3040	649.3042	$B_{3\beta}$	-0.31
1	651.2829	651.2835	$C_{3\beta}^{\bullet}$-CH_3	-0.92

z	Exp. *m/z*	Theo. *m/z*	Assignment	Δ, ppm
1	663.2839	663.2835	$B_{3\alpha}/^{1,5}X_{4\alpha''}$	0.60
1	665.2989	665.2991	$C_{3\beta}^{\ddagger}$	-0.30
1	667.3112	667.3148	$C_{3\beta}$	-5.39
1	679.2783	679.2784	$C_{3\alpha}^{\ddagger}/^{1,5}X_{4\alpha''}$	-0.15
1	695.3087	695.3097	$Y_{4\alpha''}/^{0,4}A_{4\alpha}$	-1.44
2	700.8468	700.8470	$Y_{4\alpha}$	-0.29
2	714.8453	714.8444	$^{1,5}X_{3\alpha}$	1.26
1	723.3408	723.3410	$^{1,3}A_{4}$	-0.28
2	728.8606	728.8601	$^{0,2}X_{3\alpha}$	0.69
1	734.3793	734.3792	$Y_{3\alpha}/Z_{3\beta}$	0.14
1	750.3702	750.3741	$Y_{3\alpha}/Y_{3\beta}^{\ddagger}$	-5.20
2	757.8521	757.8517	$C_{5}/Z_{3\beta}$	0.53
2	786.8855	786.8838	$Y_{4\alpha'}/Z_{4\alpha''}$	2.16
1	839.3894	839.3883	$C_{4}/Z_{3\alpha}$	1.31
1	855.3811	855.3832	$C_{4}^{\ddagger}/Y_{3\alpha}$	-2.46
1	857.4013	857.3989	$C_{4}/Y_{3\alpha}$	2.80
1	883.3739	883.3781	$C_{4}^{\ddagger}/^{1,5}X_{3\alpha}$	-4.75
1	885.3902	885.3938	$C_{4}/^{1,5}X_{3\alpha}$	-4.07
2	888.9343	888.9336	$Y_{5\alpha'}/Z_{4\alpha''}$	0.79
2	903.9388	903.9389	$Y_{3\beta}^{\ddagger}$	-0.11
2	918.9462	918.9442	$^{1,5}X_{3\beta}$	2.18
2	933.4381	933.4408	$^{1,5}A_{4}$	-2.89
2	956.4430	956.4435	$C_{4}^{\ddagger}$	-0.52
2	990.9826	990.9835	$Y_{5\alpha'}/Z_{5\alpha''}$	-0.91
2	992.4792	992.4722	$^{3,5}A_{5}$	7.05
2	997.9890	997.9914	$Z_{4\alpha'', 4\alpha', 4\beta}$	-2.40
2	999.9860	999.9888	$Y_{5\alpha'}/Y_{5\alpha''}$	-2.80
2	1004.9807	1004.9810	$Y_{4\alpha'', 4\alpha', 4\beta}^{\ddagger}$	-0.30
2	1014.4872	1014.4853	$^{2,5}A_{5}$	1.87
2	1020.9941	1020.9941	$^{1,5}X_{4\alpha'', 4\alpha', 4\beta}$	0.00
1	1027.4994	1027.4932	$^{1,5}A_{3\alpha}^{\ddagger}$	6.03
1	1029.5128	1029.5088	$^{1,5}A_{3\alpha}$	3.89
2	1033.9962	1033.9908	$B_{5}/^{0,4}X_{5\alpha''}$	5.22
2	1042.9988	1042.9961	$C_{5}/^{0,4}X_{5\alpha''}$	2.59
2	1070.5182	1070.5172	$Y_{5}/^{1,5}A_{6}$	0.93
1	1075.5133	1075.5143	$C_{3\alpha}$	-0.93
2	1079.00798	1079.0067	$C_{5}^{\ddagger}$	1.19
2	1080.0137	1080.0145	C_{5}	-0.74

z	Exp. m/z	Theo. m/z	Assignment	Δ, ppm
2	1100.0396	1100.0412	$Z_{5\alpha'', 5\alpha', 5\beta}$	-1.45
2	1108.0383	1108.0387	$Y_{5\alpha'', 5\alpha', 5\beta}{}^{‡}$	-0.36
2	1115.0348	1115.0354	$^{3,5}A_6$	-0.54
1	1117.5256	1117.5249	$^{0,4}A_4$	0.63
2	1123.0441	1123.0440	$^{1,5}X_{5\alpha'', 5\alpha', 5\beta}$	0.09
2	1145.0514	1145.0571	$^{2,5}X_{5\alpha'', 5\alpha', 5\beta}$	-4.98
1	1145.5566	1145.5562	$^{3,5}A_4$	0.35
2	1152.0640	1152.0649	$^{1,4}X_{5\alpha'', 5\alpha', 5\beta}$	-0.78
2	1187.0847	1187.0853	$M^{\bullet}$-$C_2H_6O_2$	-0.51
2	1195.0834	1195.0833	$M^{\bullet}$-C_2H_5O	0.08
2	1202.0919	1202.091	$M^{\bullet}$-CH_4O	0.75
2	1210.0890	1210.09	$M^{\bullet}$-CH_4	-0.83
2	1218.1048	1218.1042	M	0.49
1	1247.5889	1247.5879	$C_4/Z_{3\beta}$	0.80
1	1316.6719	1316.6679	$Z_{3\alpha}{}^{\bullet}$-C_2H_5O	3.04
1	1330.6749	1330.6837	$Z_{3\alpha}{}^{\bullet}$-CH_3O	-6.61
1	1344.6660	1344.6629	$Y_{3\beta}{}^{‡}/Z_{4\alpha', 4\alpha''}$	2.31
1	1346.6844	1346.6786	$Z_{3\alpha}{}^{\bullet}$-CH_3	4.31
1	1360.6887	1360.6942	$Z_{3\alpha}$	-4.04
1	1376.6934	1376.6891	$Y_{3\alpha}{}^{‡}$	3.12
1	1406.7010	1406.6997	$^{1,5}X_{3\alpha}$	0.92
1	1451.6898	1451.6876	$C_4/Z_{4\alpha'', 4\alpha', 4\beta}$	1.52
1	1492.7115	1492.7142	$C_5/Z_{3\beta}$	-1.81
1	1495.6749	1495.6775	$C_4{}^{‡}/{}^{1,5}X_{4\alpha'', 4\alpha', 4\beta}$	-1.74
1	1548.7663	1548.7626	$Y_{4\alpha'}{}^{‡}/Z_{4\alpha''}$	2.39
1	1550.7785	1550.7783	$Y_{4\alpha'}/Z_{4\alpha''}$	0.13
1	1566.7705	1566.7732	$Y_{4\alpha'}/Y_{4\alpha''}$-2H	-1.69
1	1594.7623	1594.7682	$Y_{4\alpha'}{}^{‡}/{}^{1,5}X_{4\alpha''}$	-3.67
1	1624.7772	1624.7690	C_4/Z_5-CH_3O	5.04
1	1655.7870	1655.7874	C_4/Z_5	-0.24
1	1671.7834	1671.7824	$C_4{}^{‡}/Y_5$	0.63
1	1699.7776	1699.7772	$C_4{}^{‡}/{}^{1,5}X_5$	0.24
1	1738.8824	1738.8832	$Z_{3\beta}{}^{\bullet}$-CH_3O	-0.46
1	1754.8764	1754.8781	$Y_{5\alpha'}/Z_{4\alpha''}$	-0.97
1	1768.8931	1768.8937	$Z_{3\beta}$	-0.34
1	1784.8876	1784.8886	$Y_{3\beta}{}^{‡}$	-0.56
1	1814.9018	1814.8992	$^{1,5}X_{3\beta}$	1.43
1	1845.9051	1845.9079	$^{1,5}A_4$	-1.52

z	Exp. m/z	Theo. m/z	Assignment	Δ, ppm
1	1859.8872	1859.8872	$C_4^{\bullet}$-CH_3O	0.00
1	1875.8799	1875.8821	$C_4^{\ddagger\bullet}$-CH_3	-1.17
1	1889.8982	1889.8977	$C_4^{\ddagger}$	0.26
1	1928.9668	1928.9673	$Z_{4\alpha}^{\bullet}$-C_2H_5O	-0.26
1	1942.9793	1942.9830	$Z_{4\alpha}^{\bullet}$-CH_3O	-1.90
1	1958.9772	1958.9778	$Z_{4\alpha}^{\bullet}$-CH_3	-0.31
1	1972.9958	1972.9935	$Z_{4\alpha'', 4\alpha', 4\beta}$	1.17
1	1988.9870	1988.9884	$Y_{4\alpha'', 4\alpha', 4\beta}^{\ddagger}$	-0.70
1	2019.0011	2018.9990	$^{1,5}X_{4\alpha'', 4\alpha', 4\beta}$	1.04
1	2119.0337	2119.0292	B_5	2.12
1	2177.0843	2177.0933	$Z_{5\alpha'', 5\alpha',5\beta}$	-4.13
1	2193.0907	2193.0882	$Y_{5\alpha'', 5\alpha', 5\beta}^{\ddagger}$	1.16
1	2223.0972	2223.0987	$^{1,5}X_{5\alpha'', 5\alpha', 5\beta}$	-0.67
1	2413.2175	2413.2192	M	-0.70

Table S2. List of assigned fragments by EED MS/MS of deutero-reduced and permethylated $Man_9GlcNAc_2$ ($[M+2Na]^{2+}$ at *m/z* 1218.1042), acquired on the FTICR MS instrument (spectrum shown in Figure 2b). All fragments are sodium adducts unless labeled otherwise. Multiple assignments are separated by commas; the double dagger symbol (‡) indicates that the fragment has two fewer hydrogens than the canonical structures; the double prime symbol (") indicates that the fragment has two more hydrogens than the canonical structure; the dot symbol (•) indicates a radical.

z	Exp. *m/z*	Theo. *m/z*	Assignment	Δ, ppm
1	211.0936	211.0941	$B_1^{\bullet}$-CH_3O	-2.20
1	213.0728	213.0734	$C_1^{\bullet}$-C_2H_5O	-2.86
1	227.0886	227.0890	$C_1^{\bullet}$-CH_3O	-1.87
1	241.1042	241.1046	B_1	-1.72
1	243.0836	243.0839	$C^{‡}$/Y (Hex)	-1.40
1	257.0993	257.0996	$C_1^{‡}$	-1.16
1	269.1579	269.1582	$Z_1^{\bullet}$-CH_3O	-1.13
1	285.1530	285.1531	$Z_1^{\bullet}$-CH_3	-0.50
1	296.1102	296.1105	$B_5/^{1,5}X_1$	-0.77
1	299.1686	299.1687	Z_1	-0.61
1	315.1413	315.1414	$^{1,3}A_2$	-0.39
1	315.1636	315.1637	$Y_1^{‡}$	-0.28
1	317.1792	317.1793	Y_1	-0.25
1	329.1207	329.1207	$C_2^{‡}/^{1,4}X_5$	0.09
1	345.1742	345.1742	$^{1,5}X_1$	0.00
1	371.1677	371.1676	$B_{2β}/^{0,4}X_{5β}$	0.11
1	379.1340	379.1344	$^{1,4}A_2^{‡}$(2Na-H)	-0.95
1	399.1629	399.1626	$C_2^{‡\bullet}$-$C_2H_5O_2$	0.66
1	401.1785	401.1781	$C_2^{\bullet}$-$C_2H_5O_2$	1.06
1	413.1780	413.1782	$B_{3β}/Z_{5β}$	-0.61
1	415.1941	415.1939	$^{1,5}A_2^{‡}$	0.69
1	417.1735	417.1732	$C_2^{\bullet}$-C_2H_5O	0.81
1	417.2095	417.2095	$^{1,5}A_2$	0.04
1	429.1737	429.1731	$C_2^{‡\bullet}$-CH_3O	1.29
1	431.1890	431.1888	$C_2^{\bullet}$-CH_3O	0.63
1	445.2046	445.2044	B_2	0.43
1	447.1840	447.1837	$C^{‡}_{3β}/Y_{5β}$	0.60
1	461.1997	461.1993	$C_2^{‡}$	0.80
1	463.2156	463.2150	C_2	1.22
1	475.1788	475.1786	$C_{3β}^{‡}/^{1,5}X_{5β}$	0.32

z	Exp. *m/z*	Theo. *m/z*	Assignment	Δ, ppm
1	484.2741	484.2740	$Z_2^{\bullet}$-C_2H_5O	0.36
1	485.1974	485.1993	$Z_{5\beta}/^{1,3}A_{3\beta}^{\ddagger}$	-3.94
1	500.2693	500.2688	$Z_2^{\bullet}$- C_2H_5O	1.02
1	502.2850	502.2845	$Z_2^{''\bullet}$- C_2H_5O	0.97
1	505.2262	505.2255	$^{0,4}A_{3\alpha'}$	1.27
1	514.2851	514.2845	$Z_2^{\bullet}$-CH_3O	1.04
1	519.2417	519.2412	$^{1,3}A_{3\beta,\ 3\alpha''}$	0.87
1	527.2080	527.2076	$^{0,4}A_{3\alpha'}^{\ddagger}$(2Na-H)	0.82
1	530.2799	530.2794	$Z_2^{\bullet}$-CH_3	0.93
1	533.2575	533.2568	$^{3,5}A_{3\alpha'}$	1.15
1	544.2956	544.2951	Z_2	1.04
1	547.2364	547.2361	$^{0,3}A_{3\alpha'}{}^{\ddagger}$	0.53
1	560.2908	560.2900	$Y_2^{\ddagger}$	1.44
1	561.2528	561.2518	$^{1,4}A_{3\beta,\ 3\alpha''}{}^{\ddagger}$	1.88
1	562.3061	562.3056	Y_2	0.80
1	575.2685	575.2674	$B_{3\beta}/^{0,4}X_{5\beta}$	1.86
1	583.2344	583.2338	$^{1,4}A_{3\beta,\ 3\alpha''}{}^{\ddagger}$(2Na-H)	1.10
1	590.3013	590.3005	$^{1,5}X_2$	1.31
1	605.2783	605.2779	$C_{3\beta}^{\bullet}$-$C_2H_5O_2$	0.67
1	618.3313	618.3318	$^{0,2}X_2$	-0.88
1	619.2940	619.2936	$^{1,5}A_{3\beta'}{}^{\ddagger}$	0.59
1	621.2746	621.2730	$C_{3\beta}^{\bullet}$-C_2H_5O	2.61
1	621.3096	621.3093	$^{1,5}A_{3\beta}$	0.55
1	633.2742	633.2729	$C_{3\beta}^{\ddagger\bullet}$-$CH_3O$, $C_{3\alpha}^{\ddagger}/Z_{4\alpha''}$	2.02
1	635.2894	635.2885	$C_{3\beta}^{\bullet}$-CH_3O, $C_{3\alpha}/Z_{4\alpha''}$	1.29
1	649.3046	649.3042	$B_{3\beta}$	0.60
1	651.2831	651.2835	$C_{3\beta}$-CH_3	-0.50
1	665.3000	665.2991	$C_{3\beta}^{\ddagger}$	1.27
1	667.3168	667.3148	$C_{3\beta}$	3.08
1	717.2924	717.2922	$^{0,4}A_{4\alpha}$ /$Y_{4\alpha''}$(2Na-H)	0.33
1	723.3423	723.3410	$^{1,3}A_4$	1.88
1	734.3805	734.3792	$Y_{3\alpha}$ /$Z_{3\beta}$	1.72
1	809.3792	809.3778	$Y_{5\alpha}/^{1,5}A_{3\alpha}^{\ddagger}$	1.77
1	839.3889	839.3883	$C_4/Z_{3\alpha}$	0.72
1	883.3781	883.3781	$C_4^{\ddagger}/^{1,5}X_{3\alpha}$	-0.08
2	888.9358	888.9336	$Y_{5\alpha'}/Z_{4\alpha''}$	2.40
2	895.9236	895.9233	$Y_{5\alpha'}/Y_{4\alpha''}^{\ddagger}$	0.32
2	918.9447	918.9442	$^{1,5}X_{3\beta}$	0.51
1	921.3930	921.3919	$^{0,4}A_{4\alpha}$ /$Y_{5\alpha''}$(2Na-H)	1.16

z	Exp. m/z	Theo. m/z	Assignment	Δ, ppm
2	934.4513	934.4486	$^{1,5}A_4$	2.96
1	938.4814	938.4790	$Y_{3\alpha}/Z_{4\beta}$	2.63
2	956.4448	956.4435	$C_4^{\ddagger}$	1.31
2	990.9859	990.9835	$Y_{5\alpha'}/Z_{5\alpha''}$	2.40
2	1004.9823	1004.9810	$Y_{4\alpha'', 4\alpha', 4\beta}^{\ddagger}$	1.27
2	1014.4878	1014.4853	$^{2,5}A_5$	2.45
2	1020.9955	1020.9941	$^{1,5}X_{4\alpha'', 4\alpha', 4\beta}$	1.41
1	1027.4955	1027.4932	$^{1,5}A_{3\alpha}^{\ddagger}$	2.21
1	1029.5104	1029.5088	$^{1,5}A_{3\alpha}$	1.50
1	1042.5229	1042.5263	$C_5/^{0,4}X_{5\alpha''}$	-3.27
1	1043.4882	1043.4881	$C_{3\alpha}^{\bullet}$-CH_3O	0.07
2	1057.0163	1057.0117	$^{1,5}A_5$	4.28
2	1058.0026	1058.0014	$C_5^{\ddagger\bullet}$-C_2H_5O	1.17
1	1073.5022	1073.4987	$C_{3\alpha}^{\ddagger}$	3.29
1	1075.5136	1075.5143	$C_{3\alpha}$	-0.64
2	1079.0080	1079.0067	$C_5^{\ddagger}$	1.19
2	1100.0433	1100.0412	$Z_{5\alpha'', 5\alpha', 5\beta}$	1.86
2	1108.0406	1108.0378	$Y_{5\alpha'', 5\alpha', 5\beta}^{\ddagger}$	2.49
1	1117.5251	1117.5249	$^{0,4}A_4$	0.17
2	1123.0454	1123.0440	$^{1,5}X_{5\alpha'', 5\alpha', 5\beta}$	1.25
2	1137.0610	1137.0596	$^{0,2}X_{5\alpha'', 5\alpha', 5\beta}$	1.24
1	1139.5079	1139.5069	$^{0,4}A_4$(2Na-H)	0.87
1	1145.5582	1145.5562	$^{3,5}A_4$	1.80
2	1152.0680	1152.0649	$^{1,4}X_{5\alpha'', 5\alpha', 5\beta}$	2.65
2	1167.0704	1167.0702	$^{3,5}X_{5\alpha'', 5\alpha', 5\beta}$	0.19
2	1180.5843	1180.5819	M-$C_3H_7O_2$	2.05
2	1187.0885	1187.0853	M-$C_2H_6O_2$	2.63
2	1195.0850	1195.0833	M-C_2H_5O	1.43
2	1202.0923	1202.091	M-CH_4O	0.98
2	1210.0905	1210.09	M-CH_4	0.55
2	1218.1050	1218.1042	M	0.61
1	1247.5898	1247.5879	$C_4/Z_{3\beta}$	1.53
1	1330.6900	1330.6836	$Z_{3\alpha}^{\bullet}$-CH_3O	4.79
1	1360.7014	1360.6942	$Z_{3\alpha}$	5.29
1	1376.6953	1376.6891	$Y_{3\alpha}^{\ddagger}$	4.54
1	1406.7010	1406.6996	$^{1,5}X_{3\alpha}$	0.98
1	1462.7046	1462.7036	$Y_{3\beta}/^{1,5}A_5^{\ddagger}$	0.65
1	1699.7707	1699.7772	$C_4^{\ddagger/1,5}X_{4\alpha}$	-3.82
1	1738.8873	1738.8832	$Z_{3\beta}^{\bullet}$-CH_3O	2.38

z	Exp. m/z	Theo. m/z	Assignment	Δ, ppm
1	1754.8776	1754.8781	$Y_{5\alpha'}/Z_{4\alpha''}$	-0.27
1	1768.8981	1768.8937	$Z_{3\beta}$	2.49
1	1784.8851	1784.8886	$Y_{3\beta}^{‡}$	-1.97
1	1814.8979	1814.8992	$^{1,5}X_{3\beta}$	-0.70
1	1845.9077	1845.9079	$^{1,5}A_{5}$	-0.10
1	1875.8810	1875.8821	$C_{4}^{‡}$-CH_3	-0.61
1	1889.8974	1889.8977	$C_{4}^{‡}$	-0.18
1	1928.9689	1928.9672	$Z_{4\alpha'', 4\alpha', 4\beta}^{\bullet}$-$C_2H_5O$	0.87
1	1942.9802	1942.9829	$Z_{4\alpha'', 4\alpha', 4\beta}^{\bullet}$-$CH_3O$	-1.41
1	1958.9777	1958.9778	$Y_{5\alpha'}/Z_{5\alpha''}$	-0.05
1	1972.9840	1972.9935	$Z_{4\alpha'', 4\alpha', 4\beta}$	-4.81
1	2019.0035	2018.9990	$^{1,5}X_{4\alpha'', 4\alpha', 4\beta}$	2.26
1	2119.0266	2119.0292	B_5	-1.20
1	2223.0984	2223.0987	$^{1,5}X_{5\alpha'', 5\alpha', 5\beta}$	-0.15
1	2337.1693	2337.1668	M-$C_3H_8O_2$	1.05
1	2381.1948	2381.1930	M-CH_4O	0.73
1	2413.2185	2413.2192	M	-0.30

Table S3. List of assigned fragments by CID-EED MS^3 of the $^{0,4}A_4$ ion from deutero-reduced and permethylated $Man_9GlcNAc_2$ at *m/z* 1117.5259, acquired on the QE-Omnitrap platform (spectrum shown in Figure 4b). All fragments are sodium adducts unless labeled otherwise. Multiple assignments are separated by commas; the double dagger symbol (‡) indicates that the fragment has two fewer hydrogens than the canonical structures; the double prime symbol (") indicates that the fragment has two more hydrogens than the canonical structure; the dot symbol (•) indicates a radical.

z	Exp. *m/z*	Theo. *m/z*	Assignment	Δ, ppm
1	227.0893	227.0890	$C_1^{\bullet}$-CH_3O	1.35
1	243.0844	243.0839	$C^{‡}$/Y (Hex)	1.88
1	255.0841	255.0839	$Y_{4\alpha'}/Z_{4\alpha''}/^{0,4}A_4$	0.55
1	259.1162	259.1152	C_1	3.73
1	315.1053	315.1050	$C_2^{‡}/^{2,5}X_5$	0.73
1	315.1416	315.1414	$^{1,3}A_2$	0.57
1	343.1369	343.1363	$B_2/^{3,5}X_5$	1.51
1	417.1737	417.1732	$C_2^{\bullet}$-C_2H_5O	1.26
1	431.1890	431.1888	$C_2^{\bullet}$-CH_3O	0.59
1	443.1902	443.1888	$B_2^{‡}$	3.32
1	445.2045	445.2044	B_2	0.29
1	459.1841	459.1837	$Y_{5\alpha'}/Z_{4\alpha''}/^{0,4}A_4$	0.83
1	461.1997	461.1993	$C_2^{‡}$	0.78
1	463.2153	463.2150	C_2	0.60
1	505.2262	505.2255	$^{0,4}A_{3\alpha'}$	1.22
1	519.2413	519.2412	$^{1,3}A_{3\alpha''}$	0.23
1	533.2575	533.2568	$^{3,5}A_{3\alpha'}$	1.30
1	633.2760	633.2768	$Z_{4\alpha}^{\bullet}$-$C_2H_5O/^{0,4}A_4$	-1.38
1	635.2889	635.2885	$C_{3\alpha}/Z_{4\alpha''}$	0.57
1	647.2894	647.2926	$Z_{4\alpha}^{\bullet}$-$CH_3O/^{0,4}A_4$	-4.98
1	663.2847	663.2875	$Y_{5\alpha'}/Z_{5\alpha''}/^{0,4}A_4$	-4.25
1	677.2997	677.3031	$Z_{4\alpha}/^{0,4}A_4$	-5.04
1	693.2953	693.2981	$Y_{4\alpha}^{‡}/^{0,4}A_4$	-4.04
1	695.3094	695.3137	$Y_{4\alpha}/^{0,4}A_4$	-6.23
1	723.3057	723.3086	$^{1,5}X_{4\alpha}/^{0,4}A_4$	-3.99
1	781.3448	781.3465	$Y_{5\alpha}^{‡}/^{0,2}A_3$	-2.06
1	897.3945	897.3978	$Y_{5\alpha}^{‡}/^{0,4}A_4$	-3.70
1	927.4044	927.4084	$^{1,5}X_{5\alpha}/^{0,4}A_4$	-4.26
1	955.4356	955.4397	$^{0,2}X_{5\alpha}/^{0,4}A_4$	-4.25
1	985.4462	985.4503	$^{1,4}X_{5\alpha}/^{0,4}A_4$	-4.15

z	Exp. m/z	Theo. m/z	Assignment	Δ, ppm
1	1117.5259	1117.5289	M	-2.68

Table S4. List of assigned fragments by CID-EED MS^3 of the $B_{3\beta}$ ion from deutero-reduced and permethylated $Man_9GlcNAc_2$ at *m/z* 649.3046, acquired on the QE-Omnitrap platform (spectrum shown in Figure 4c). All fragments are sodium adducts unless labeled otherwise. Multiple assignments are separated by commas; the double dagger symbol (‡) indicates that the fragment has two fewer hydrogens than the canonical structures; the double prime symbol (") indicates that the fragment has two more hydrogens than the canonical structure; the dot symbol (•) indicates a radical.

z	Exp. *m/z*	Theo. *m/z*	Assignment	Δ, ppm
1	225.0733	225.0734	$C_1^{‡•}$-CH_3O	-0.44
1	227.0890	227.0890	$C_1^{•}$-CH_3O	0.00
1	241.1046	241.1046	B_1	0.00
1	243.0838	243.0839	$C^‡$/Y (Hex)	-0.41
1	255.0838	255.0839	$^{1,5}X_4/B_{3\beta}$	-0.39
1	257.0993	257.0996	$C_1^‡$	-1.17
1	259.1153	259.1152	C_1	0.39
1	311.1098	311.1101	$^{1,4}X_2^‡$	-0.96
1	313.1256	313.1258	$^{1,3}A_2^‡$	-0.64
1	315.1415	315.1414	$^{1,3}A_2$	0.32
1	345.1521	345.1520	$C_{2\beta}/^{0,3}X_{5\beta}$	0.29
1	353.1568	353.1571	$Z_{5\beta}^{•}$-$C_2H_5O_2/B_{3\beta}$	-0.85
1	361.1468	361.1469	$C_{2\beta}/^{3,5}X_{5\beta}$	-0.28
1	369.1513	369.1519	$Z_{5\beta}^{•}$-$C_2H_5O/B_{3\beta}$	-1.63
1	383.1676	383.1676	$Z_{5\beta}/^{1,5}A_3^‡$	0.00
1	385.1468	385.1469	$Z_{5\beta}^{•}$-$CH_3O/B_{3\beta}$	-0.26
1	399.1624	399.1625	$Z_{5\beta}^{•}$-$CH_3/B_{3\beta}$	-0.25
1	413.1782	413.1782	$Z_{5\beta}/B_{3\beta}$	0.00
1	415.1939	415.1939	$^{1,5}A_2^‡$	0.00
1	429.1733	429.1731	$Y_{5\beta}^‡/B_{3\beta}$	0.47
1	431.1887	431.1887	$Y_{5\beta}/B_{3\beta}$	0.00
1	445.2043	445.2044	B_2	-0.22
1	459.1838	459.1836	$^{1,5}X_{5\beta}/B_{3\beta}$	0.44
1	461.1999	461.1993	$C_2^‡$	1.30
1	463.2153	463.2150	C_2	0.65
1	487.2149	487.2149	$^{0,2}X_{5\beta}/B_{3\beta}$	0.00
1	515.2094	515.2099	$^{1,4}X_{5\beta}^‡/B_{3\beta}$	-0.97
1	517.2259	517.2255	$^{1,3}A_3^‡$	0.77
1	519.2419	519.2412	$^{1,3}A_3$	1.35
1	573.2516	573.2517	M-$C_3H_7O_2$	-0.17

z	Exp. m/z	Theo. m/z	Assignment	Δ, ppm
1	587.2669	587.2674	M-$C_2H_5O_2$	-0.85
1	603.2614	603.2624	M-C_2H_5O	-1.66
1	617.2783	617.2780	M-CH_3O	0.49
1	649.3041	649.3042	M	-0.15

Table S5. List of assigned fragments by CID-EED MS^3 of the $Y_{3\beta}^{2+}$ ion from deutero-reduced and permethylated $Man_9GlcNAc_2$ at *m/z* 904.9469, acquired on QE-Omnitrap MS (spectrum shown in Figure S1). All fragments are sodium adducts unless labeled otherwise. Multiple assignments are separated by commas; the double dagger symbol (‡) indicates that the fragment has two fewer hydrogens than the canonical structures; the double prime symbol (") indicates that the fragment has two more hydrogens than the canonical structure; the dot symbol (•) indicates a radical.

z	Exp. *m/z*	Theo. *m/z*	Assignment	Δ, ppm
1	227.0889	227.0890	$C_{1\alpha}^{\bullet}$-OCH_3	-0.44
1	241.1045	241.1046	$B_{1\alpha}$	-0.41
1	255.1424	255.1425	$Z_1^{\bullet}$-OC_2H_5	-0.39
1	257.0994	257.0996	$C_{1\alpha}^{‡}$	-0.78
1	259.1145	259.1152	$C_{1\alpha}$	-2.70
1	269.1580	269.1582	$Z_1^{\bullet}$-OCH_3	-0.74
1	285.1529	285.1531	$Z_1^{\bullet}$-CH_3	-0.70
1	299.1685	299.1687	Z_1	-0.67
1	315.1411	315.1414	$^{3,5}A_3$	-0.95
1	317.1792	317.1793	Y_1	-0.32
1	339.1609	339.1613	Y_1(2Na-H)	-1.18
1	345.1741	345.1742	$^{1,5}X_1$	-0.29
1	385.1831	385.1830	$B_{2\alpha}^{\bullet}$-$O_2C_2H_5$	0.26
1	386.2004	386.2006	$^{1,5}A_{2\alpha}$-OCH_3	-0.52
1	379.1338	379.1340	$^{1,4}A_{2\alpha}^{‡}$(2Na-H)	-0.53
1	401.1782	401.1782	$C_{2\alpha}^{\bullet}$-$O_2C_2H_5$	0.00
1	414.2325	414.2321	$^{0,2}X_1$	0.97
1	417.2090	417.2095	$^{1,5}A_{2\alpha}$	-1.20
1	431.1887	431.1888	$C_{2\alpha}^{\bullet}$-OCH_3	-0.23
1	445.2043	445.2044	$B_{2\alpha}$	-0.22
1	461.1992	461.1994	$C_{2\alpha}^{‡}$	-0.43
1	463.2145	463.2150	$C_{2\alpha}$	-1.08
1	500.2684	500.2689	$Z_2^{\bullet}$-OC_2H_5	-1.00
1	505.2251	505.2255	$^{0,4}A_{3\alpha}$	-0.79
1	514.2845	514.2846	$Z_2^{\bullet}$-OCH_3	-0.19
1	519.2409	519.2412	$^{1,3}A_{3\alpha}$	-0.58
1	527.2076	527.2075	$^{0,4}A_{3\alpha}$(2Na-H)	0.19
1	530.2792	530.2795	$Z_2^{\bullet}$-CH_3	-0.57
1	533.2572	533.2568	$^{3,5}A_{3\alpha}$	0.75

z	Exp. *m/z*	Theo. *m/z*	Assignment	Δ, ppm
1	544.2950	544.2951	Z_2	-0.18
1	562.3057	562.3056	Y_2	0.18
1	590.3008	590.3006	$^{1,5}X_2$	0.34
1	618.3317	618.3319	$^{0,2}X_2$	-0.32
1	634.3256	634.3268	$^{2,5}X_2$	-1.89
1	635.2886	635.2885	$C_{3\alpha}/Z_{4\alpha''}$	0.16
2	644.2909	644.2938	$C_4/Y_{4\beta}$	-4.50
2	662.8192	662.8208	$Z_{4\alpha}^{\bullet}$-$OC_2H_5/Y_{4\beta}$	-2.41
2	669.8288	669.8286	$Z_{4\alpha}^{\bullet}$-$OCH_3/Y_{4\beta}$	0.30
2	677.8255	677.8261	$Z_{4\alpha}^{\bullet}$-$CH_3/Y_{4\beta}$	-0.89
2	684.8315	684.8339	$Z_{4\alpha}/Y_{4\beta}$	-3.50
1	690.3537	690.3530	$Z_{3\alpha}^{\bullet}$-$OC_2H_5/Y_{4\beta}$	1.01
2	693.8374	693.8392	$Y_{4\alpha}/Y_{4\beta}$	-2.59
1	704.3681	704.3687	$Z_{3\alpha}^{\bullet}$-$OCH_3/Y_{4\beta}$	-0.85
2	707.8368	707.8366	$^{1,5}X_{4\alpha}/Y_{4\beta}$	0.28
1	720.3644	720.3636	$Z_{3\alpha}^{\bullet}$-$CH_3/Y_{4\beta}$	1.11
1	734.3798	734.3792	$Z_{3\alpha}/Y_{4\beta}$	0.82
1	742.3475	742.3456	$Z_{3\alpha}^{\bullet}$-$CH_3/Y_{4\beta}$ (2Na-H)	2.56
2	743.8544	743.8542	$^{1,5}A_5/Y_{4\beta}$	0.27
2	750.8434	750.8439	$C_5^{\bullet}$-$OCH_3/Y_{4\beta}$	-0.67
1	752.3870	752.3898	$Y_{3\alpha}/Y_{4\beta}$	-3.72
2	757.8518	757.8517	$B_5/Y_{4\beta}$	0.13
2	764.8695	764.8706	$Z_{5\alpha}^{\bullet}$-$OC_2H_5/Y_{4\beta}$	-1.44
2	765.8484	765.8492	$C_5^{\ddagger}/Y_{4\beta}$	-1.04
2	766.8559	766.8570	$C_5/Y_{4\beta}$	-1.43
2	771.8776	771.8785	$Z_{5\alpha}^{\bullet}$-$OCH_3/Y_{4\beta}$	-1.17
1	774.3722	774.3718	$Y_{3\alpha}/Y_{4\beta}$(2Na-H)	0.52
2	779.8750	779.8759	$Z_{5\alpha}^{\bullet}$-$CH_3/Y_{4\beta}$	-1.15
1	780.3832	780.3847	$^{1,5}X_{3\alpha}/Y_{4\beta}$	-1.92
2	786.8829	786.8837	$Z_{5\alpha}/Y_{4\beta}$	-1.02
2	795.8863	795.8890	$Y_{5\alpha}/Y_{4\beta}$	-3.39
2	809.8866	809.8865	$^{1,5}X_{5\alpha}/Y_{4\beta}$	0.12
2	823.9005	823.9021	$^{0,2}X_{5\alpha}/Y_{4\beta}$	-1.94
2	831.8890	831.8885	$^{0,2}A_6/Y_{4\beta}$	0.60
2	838.9056	838.9074	$^{1,4}X_{5\alpha}/Y_{4\beta}$	-2.15
2	853.9135	853.9127	$^{3,5}X_{5\alpha}/Y_{4\beta}$	0.94
2	866.4159	866.4174	$^{1,5}A_6/Y_{4\beta}$	-1.73
2	867.9275	867.9284	$^{0,4}X_{5\alpha}/Y_{4\beta}$	-1.04

z	Exp. m/z	Theo. m/z	Assignment	Δ, ppm
2	881.9251	881.9260	$M\text{-}OC_2H_5$	-0.98
2	888.9335	888.9338	$M\text{-}OCH_3$	-0.33
2	896.9310	896.9313	$M\text{-}CH_4$	-0.28
2	904.9469	904.9467	M	0.22
1	1029.5095	1029.5088	$^{1,5}A_{3\alpha}$	0.68
1	1043.4892	1043.4881	$C_{3\alpha}^{\bullet}\text{-}OCH_3$	1.05
1	1073.5020	1073.4987	$C_{3\alpha}^{\ddagger}$	3.07
1	1075.5155	1075.5143	$C_{3\alpha}$	1.12
1	1117.5231	1117.5249	$^{0,4}A_4$	-1.61
1	1139.5064	1139.5069	$^{0,4}A_4$(2Na-H)	-0.44
1	1145.5592	1145.5562	$^{3,5}A_4$	2.62
1	1233.5720	1233.5722	$C_4^{\bullet}\text{-}OCH_3/Y_{4\beta}$	-0.16
1	1247.5872	1247.5879	$B_4/Y_{4\beta}$	-0.56
1	1263.5831	1263.5828	$C_4^{\ddagger}/Y_{4\beta}$	0.24
1	1265.5941	1265.5984	$C_4/Y_{4\beta}$	-3.40
1	1316.6666	1316.6680	$Z_{4\alpha}^{\bullet}\text{-}OCH_3/Y_{4\beta}$	-1.06
1	1332.6622	1332.6629	$Z_{4\alpha}^{\bullet}\text{-}CH_3/Y_{4\beta}$	-0.53
1	1335.6429	1335.6403	$^{3,5}A_5/Y_{4\beta}$	1.95
1	1346.6762	1346.6785	$Z_{4\alpha}/Y_{4\beta}$	-1.71
1	1392.6847	1392.6840	$^{1,5}X_{4\alpha}/Y_{4\beta}$	0.50
1	1466.6985	1466.6982	$C_5^{\bullet}\text{-}OCH_3/Y_{4\beta}$	0.20
1	1492.7148	1492.7142	$B_5/Y_{4\beta}$	0.40
1	1508.7069	1508.7091	$C_5^{\ddagger}/Y_{4\beta}$	-1.46
1	1786.8950	1786.9043	M	-5.20

Table S6. List of assigned fragments by EED MS^2 of deutero-reduced and permethylated G2 ($Hex_5HexNAc_4$, $[M+2Na]^{2+}$ at *m/z* 1055.0308), acquired on QE-Omnitrap MS (spectrum shown in Figure S4). All fragments are sodium adducts unless labeled otherwise. Multiple assignments are separated by commas; the double dagger symbol (‡) indicates that the fragment has two fewer hydrogens than the canonical structures; the double prime symbol (") indicates that the fragment has two more hydrogens than the canonical structure; the dot symbol (•) indicates a radical.

z	Exp. *m/z*	Theo. *m/z*	Assignment	Δ, ppm
1	181.0834	181.0835	$B_1^{\bullet}$-$C_2H_5O_2$	-0.55
1	197.0785	197.0784	$C_1^{\bullet}$-$C_2H_5O_2$	0.51
1	211.0941	211.0941	$B_1^{\bullet}$-OCH_3	0.00
1	227.0890	227.0890	$C_1^{\bullet}$-OCH_3	0.00
1	239.1476	239.1471	$Z_1^{\bullet}$-$C_2H_5O_2$	2.09
1	241.1047	241.1046	B_1	0.23
1	243.0841	243.0839	$C_1^{\bullet}$-CH_3	0.82
1	255.1427	255.1420	$Z_1^{\bullet}$-C_2H_5O	2.74
1	259.1152	259.1152	C_1	0.00
1	269.1582	269.1577	$Z_1^{\bullet}$-OCH_3	1.86
1	277.1868	277.1863	Z_1 (H)	1.89
1	285.1531	285.1526	$Z_1^{\bullet}$-CH_3	1.75
1	299.1687	299.1682	Z_1	1.60
1	315.1414	315.1414	$^{2,4}A_2$	0.00
1	317.1793	317.1788	Y_1	1.62
1	329.1570	329.1571	$^{3,5}A_2$	-0.23
1	345.1742	345.1737	$^{1,5}X_1$	1.45
1	357.1517	357.1520	$^{2,5}A_2$-CH_3	-0.84
1	396.1992	396.1993	$^{1,5}A_2$-$C_2H_5O_2$	-0.25
1	400.1955	400.1942	$^{1,4}A_2$	3.28
1	412.1942	412.1943	$^{1,5}A_2$-C_2H_5O	-0.24
1	414.2321	414.2316	$^{0,2}X_1$	1.33
1	426.2098	426.2099	$^{1,5}A_2$-OCH_3	-0.23
1	442.2049	442.2047	$C_2^{\bullet}$-$C_2H_5O_2$	0.45
1	458.2001	458.1997	$C_2^{\bullet}$-C_2H_5O	0.87
1	458.2363	458.2361	$^{1,5}A_2$	0.54
1	464.2490	464.2490	B_2(H)	0.00
1	472.2156	472.2153	$C_2^{\bullet}$-OCH_3	0.64

z	Exp. *m/z*	Theo. *m/z*	Assignment	Δ, ppm
1	484.2741	484.2734	$Z_2^{\bullet}$-$C_2H_5O_2$	1.45
1	486.2311	486.2310	B_2	0.27
1	488.2105	488.2102	$C_2^{\bullet}$-CH_3	0.61
1	488.2674	488.2683	$^{2,4}X_1$	-1.90
1	500.2687	500.2683	Z_2-C_2H_5O	0.80
1	502.2258	502.2274	$C_2^{\ddagger}$	-3.19
1	504.2430	504.2415	C_2	2.91
1	514.2845	514.2840	$Z_2^{\bullet}$-OCH_3	0.97
1	530.2793	530.2789	$Z_2^{\bullet}$-CH_3	0.75
1	544.2953	544.2945	Z_2	1.39
1	546.2745	546.2738	$Y_2^{\bullet}$-CH_3	1.28
1	560.2679	560.2677	$^{1,3}A_3$	0.27
1	562.3059	562.3051	Y_2	1.41
1	590.3008	590.3000	$^{1,5}X_2$	1.31
1	600.2987	600.2990	$^{1,5}A_3$-$C_2H_5O_2$	-0.50
1	604.2945	604.2940	$^{1,4}A_3$	0.89
1	618.3325	618.3313	$^{0,2}X_2$	1.90
1	630.3101	630.3096	$^{1,5}A_3$-OCH_3	0.79
1	646.3043	646.3045	$C_3^{\bullet}$-$C_2H_5O_2$	-0.31
1	662.2999	662.2995	$C_3^{\bullet}$-C_2H_5O	0.60
1	662.3358	662.3358	$^{1,5}A_3$	0.00
1	676.3150	676.3151	$C_3^{\bullet}$-OCH_3	-0.15
1	686.3601	686.3594	$C_3(H)$	1.08
1	692.3101	692.3100	$C_3^{\bullet}$-CH_3	0.14
1	706.3257	706.3257	$C_3^{\ddagger}$	0.00
1	708.3378	708.3413	C_3	-4.95
2	712.3561	712.3547	Z_3	1.96
2	721.3602	721.3600	Y_3	0.30
2	735.3541	735.3574	$^{1,5}X_3$	-4.55
1	750.3516	750.3519	$^{0,4}A_4$	-0.36
1	764.3711	764.3675	$^{1,3}A_4$	4.68
1	778.3844	778.3832	$^{3,5}A_4$	1.58
2	793.3699	793.3703	$C_4^{\ddagger}$	-0.45
2	814.4061	814.4046	Z_4	1.85
2	823.4098	823.4099	Y_4	-0.09
2	829.3996	829.3990	$^{3,5}A_5$	0.70
2	837.4079	837.4073	$^{1,5}X_4$	0.68
2	866.4322	866.4283	$^{1,4}X_4$	4.54
2	871.9345	871.9363	$^{0,2}X_4$	-2.01

z	Exp. m/z	Theo. m/z	Assignment	Δ, ppm
1	880.4153	880.4149	$C_4/Z_{3\beta}$	0.45
2	893.9418	893.9385	$^{1,5}A_5$	3.68
2	896.9442	896.9450	B_5(H+Na)	-0.88
2	905.9478	905.9503	C_5(H+Na)	-2.73
2	914.9546	914.9552	$Z_5^{\bullet}$-C_2H_5O	-0.66
2	915.9315	915.9334	$C_5^{\ddagger}$	-2.07
2	921.9628	921.9631	$Z_5^{\bullet}$-OCH_3	-0.33
2	929.9603	929.9605	$Z_5^{\bullet}$-CH_3	-0.22
2	936.9680	936.9678	Z_5	0.26
2	944.9645	944.9652	$Y_5^{\ddagger}$	-0.74
2	951.9609	951.9622	$^{3,5}A_6$	-1.34
2	959.9706	959.9705	$^{1,5}X_5$	0.11
2	973.9826	973.9861	$^{0,2}X_5$	-3.64
2	981.9756	981.9727	$^{0,2}A_6$	2.91
2	988.9913	988.9914	$^{1,4}X_5$	-0.13
2	1016.5006	1016.5017	$^{1,5}A_6$	-1.05
2	1018.0114	1018.0124	$^{0,4}X_5$	-0.94
2	1033.0168	1033.0176	M-C_2H_4O	-0.77
2	1039.0178	1039.0176	M-CH_4O	0.19
2	1055.0308	1055.0307	M	0.09
1	1100.5094	1100.5090	$C_4/Y_4^{\ddagger}$	0.38
1	1125.5409	1125.5412	C_5/Z_3	-0.27
1	1160.5901	1160.5888	$Y_{4\alpha}/Y_{4\beta}$	1.12
1	1188.5830	1188.5837	$^{1,5}X_{4\alpha}/Y_{4\beta}$	-0.59
1	1329.6368	1329.6410	C_5/Z_4	-3.16
1	1357.6995	1357.6940	$Z_3^{\bullet}$-C_2H_5O	4.05
1	1371.7117	1371.7097	$Z_3^{\bullet}$-OCH_3	1.46
1	1387.7055	1387.7046	$Z_{4\alpha}/Y_{5\beta}$	0.65
1	1401.7226	1401.7202	Z_3	1.72
1	1417.7161	1417.7152	$Y_3^{\ddagger}$	0.67
1	1419.7264	1419.7308	Y_3	-3.07
1	1447.7265	1447.7257	$^{1,5}X_3$	0.58
1	1519.7606	1519.7615	$^{1,5}A_4$	-0.57
1	1563.7497	1563.7513	$C_4^{\ddagger}$	-1.02
1	1565.7600	1565.7669	C_4	-4.44
1	1605.8217	1605.8200	Z_4	1.08
1	1623.8292	1623.8305	Y_4	-0.82
1	1635.8058	1635.8088	$^{3,5}A_5$	-1.84
1	1651.8276	1651.8254	$^{1,5}X_4$	1.31

z	Exp. m/z	Theo. m/z	Assignment	Δ, ppm
1	1766.8697	1766.8671	$C_5''^{\bullet}$-OCH_3	1.47
1	1792.8719	1792.8827	B_5	-6.03
1	2013.0342	2013.0355	$^{0,4}X_5$	-0.64
1	2087.0753	2087.0723	M	1.44

Table S7. List of assigned fragments by HCD-EED MS^3 experiment of the Y_4 ion from deutero-reduced and permethylated $Hex_9GlcNAc_2$ at *m/z* 904.9469, acquired on a QE-Omnitrap platform (spectrum shown in Figure S5b). All fragments are sodium adducts unless labeled otherwise. Multiple assignments are separated by commas; the double dagger symbol (‡) indicates that the fragment has two fewer hydrogens than the canonical structures; the double prime symbol (") indicates that the fragment has two more hydrogens than the canonical structure; the dot symbol (•) indicates a radical.

z	Exp. *m/z*	Theo. *m/z*	Assignment	Δ, ppm
1	227.0888	227.0890	$C_{1\beta}^{\bullet}$-OCH_3	-0.88
1	245.0997	245.0996	$C_{1\alpha}/Y_{4\alpha}$	0.41
1	259.1149	259.1152	$C_{1\beta}$	-1.19
1	277.1868	277.1868	Z_1 (H)	0.00
1	299.1676	299.1682	Z_1	-2.08
1	317.1786	317.1788	Y_1	-0.59
1	329.1566	329.1571	$^{3,5}A_{2\beta}$	-1.44
1	432.2229	432.2228	$B_{3\beta}/Z_{5\beta}$ (H)	0.23
1	464.2492	464.2490	$B_{2\beta}$ (H)	0.43
1	486.2311	486.2310	$B_{2\beta}$	0.27
1	504.2427	504.2415	$C_{2\beta}$	2.32
1	544.2953	544.2945	Z_2	1.39
1	560.2685	560.2692	$^{1,3}A_{3\beta}$	-1.25
1	562.3042	562.3051	Y_2	-1.62
1	590.3008	590.3000	$^{1,5}X_2$	1.31
2	634.8070	634.8074	$^{1,4}X_{4\beta}/Y_{4\alpha}$	-0.61
1	662.2998	662.2994	$Z_{3\beta}/C_5/Y_{4\alpha}$	0.60
1	690.3295	690.3307	$B_{3\beta}$	-1.74
2	694.3564	694.3562	$Z_{5\beta}/Y_{4\alpha}$ (H+Na)	0.29
1	706.3257	706.3257	$C_{3\beta}^{‡}$	0.06
1	734.3794	734.3787	$Y_{3\beta}/Z_{3\alpha}$	0.95
1	750.3511	750.3519	$^{0,2}A_6/Y_{4\alpha}$	-1.03
1	866.3987	866.3992	$Z_{5\beta}/C_4/Y_{4\alpha}$	-0.58
1	880.4156	880.4149	$Z_{3\alpha}/C_4/Y_{4\alpha}$	0.80
1	938.4795	938.4784	$Z_{3\beta}/Y_{4\alpha}$	1.13
1	956.4879	956.4890	$Y_{3\beta}/Y_{4\alpha}$	-1.16
1	984.4846	984.4839	$^{1,5}X_{3\beta}/Y_{4\alpha}$	0.69
1	1056.5216	1056.5197	$^{1,5}A_4/Y_{4\alpha}$	1.78
1	1100.5093	1100.5096	$C_4^{‡}/Y_{4\alpha}$	-0.23

z	Exp. *m/z*	Theo. *m/z*	Assignment	Δ, ppm
1	1142.5772	1142.5782	$Z_{4\beta}/Y_{4\alpha}$	-0.89
1	1158.5740	1158.5737	$Y_{4\beta}{}^{‡}/Y_{4\alpha}$	0.26
1	1160.5867	1160.5888	$Y_{4\beta}/Y_{4\alpha}$	-1.79
1	1172.5648	1172.5671	$^{3,5}A_5/Y_{4\alpha}$	-1.93
1	1188.5844	1188.5837	$^{1,5}X_{4\beta}/Y_{4\alpha}$	0.59
1	1246.6245	1246.6256	$^{1,4}X_{4\beta}/Y_{4\alpha}$	-0.85
1	1301.6460	1301.6460	$^{1,5}A_5/Y_{4\alpha}$	0.00
1	1303.6250	1303.6254	$C_5{}^{''\bullet}$-$OCH_3/Y_{4\alpha}$	-0.27
1	1329.6444	1329.6410	$B_5/Y_{4\alpha}$	2.59
1	1347.6545	1347.6515	$C_5/Y_{4\alpha}$	2.21
1	1387.7053	1387.7045	$Z_{5\beta}/Y_{4\alpha}$	0.55
1	1401.7217	1401.7202	$Z_{3\alpha}$	1.08
1	1405.7167	1405.7151	$Y_{5\beta}/Y_{4\alpha}$	1.14
1	1419.7288	1419.7308	$Y_{3\alpha}$	-1.38
1	1433.7104	1433.7100	$^{1,5}X_{5\beta}/Y_{4\alpha}$	0.27
1	1447.7269	1447.7257	$^{1,5}X_{3\alpha}$	0.85
1	1477.7163	1477.7145	$^{0,2}A_6/Y_{4\alpha}$	1.20
1	1549.7931	1549.7937	$^{1,3}X_{3\alpha}$	-0.42
1	1591.8040	1591.8043	$Y_{4\alpha}{}^{\bullet}$-OCH_4	-0.21
1	1623.8269	1623.8305	$Y_{4\alpha}$	-2.23
